# Direct Monitoring of the Strand Passage Reaction of DNA Topoisomerase II Triggers Checkpoint Activation

**DOI:** 10.1371/journal.pgen.1003832

**Published:** 2013-10-03

**Authors:** Katherine L. Furniss, Hung-Ji Tsai, Jo Ann W. Byl, Andrew B. Lane, Amit C. Vas, Wei-Shan Hsu, Neil Osheroff, Duncan J. Clarke

**Affiliations:** 1 Department of Genetics, Cell Biology & Development, University of Minnesota, Minneapolis, Minnesota, United States of America; 2 Department of Biochemistry, Vanderbilt University School of Medicine, Nashville, Tennessee, United States of America; Stowers Institute for Medical Research, United States of America

## Abstract

By necessity, the ancient activity of type II topoisomerases co-evolved with the double-helical structure of DNA, at least in organisms with circular genomes. In humans, the strand passage reaction of DNA topoisomerase II (Topo II) is the target of several major classes of cancer drugs which both poison Topo II and activate cell cycle checkpoint controls. It is important to know the cellular effects of molecules that target Topo II, but the mechanisms of checkpoint activation that respond to Topo II dysfunction are not well understood. Here, we provide evidence that a checkpoint mechanism monitors the strand passage reaction of Topo II. In contrast, cells do not become checkpoint arrested in the presence of the aberrant DNA topologies, such as hyper-catenation, that arise in the absence of Topo II activity. An overall reduction in Topo II activity (i.e. slow strand passage cycles) does not activate the checkpoint, but specific defects in the T-segment transit step of the strand passage reaction do induce a cell cycle delay. Furthermore, the cell cycle delay depends on the divergent and catalytically inert C-terminal region of Topo II, indicating that transmission of a checkpoint signal may occur via the C-terminus. Other, well characterized, mitotic checkpoints detect DNA lesions or monitor unattached kinetochores; these defects arise *via* failures in a variety of cell processes. In contrast, we have described the first example of a distinct category of checkpoint mechanism that monitors the catalytic cycle of a single specific enzyme in order to determine when chromosome segregation can proceed faithfully.

## Author Summary

Several major classes of anti-cancer drugs kill tumor cells by binding to the enzyme DNA topoisomerase II, but at the same time, cellular responses are activated that protect the tumor cells. How checkpoint activation occurs under circumstances of topoisomerase II perturbation is not well understood. We show that a novel checkpoint mechanism directly monitors the enzyme reaction of topoisomerase II. This is the first example of a checkpoint mechanism that directly monitors specific steps of the catalytic cycle of a single enzyme.

## Introduction

**Figure 1:**
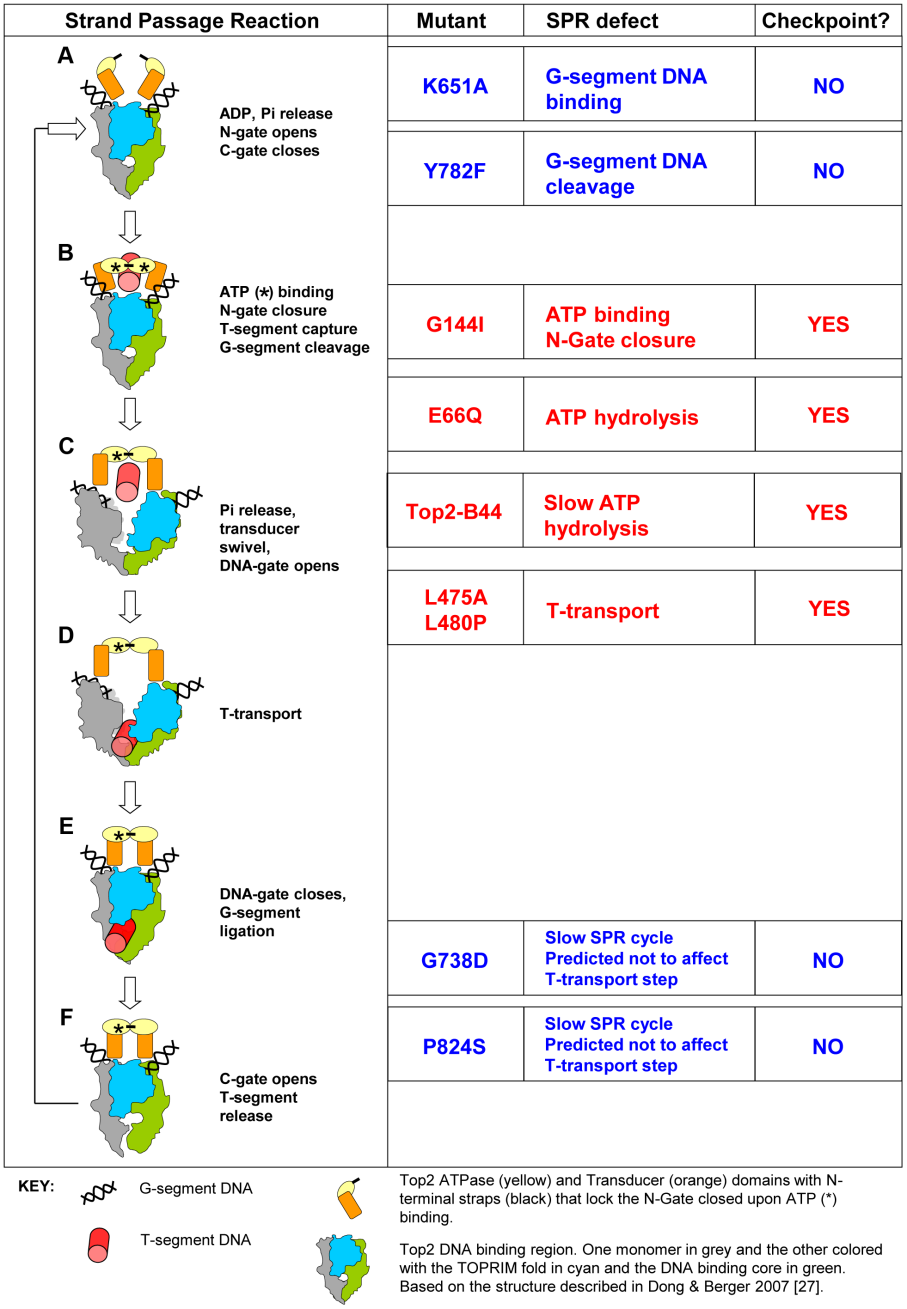
Topo II Strand Passage Reaction (SPR) and Mutants Analyzed in this Study. *Left column*, Main features of the SPR of the wild type Top2 enzyme. The cartoons and description are based on Dong and Berger 2007 and Wang 2002 [3], [27]. **a**, Top2 homodimer bound to G-segment DNA (see Key). N-Gate is open in the absence of bound nucleotide. **b**, Binding of one molecule of ATP to each monomer is required for N-Gate closure. If a T-segment is captured, then G-segment cleavage is thought to be coupled with N-Gate closure, in order for the T-segment to be accommodated. **c**–**d**, hydrolysis of one ATP promotes conformation changes that swivel the Transducer domain, opening the DNA-Gate and leading to T-transport. **e**, DNA-Gate closure leads to G-segment re-ligation. **f**, C-Gate opening allows T-segment release followed by hydrolysis of the second ATP and N-Gate opening after release of the hydrolysis products. *Mutants*: K651A has greatly reduced affinity for G-segment DNA and thus does not perform appreciable strand passage reactions *in vivo* and cannot support viability [30]. *In vitro*, relaxation of supercoiled DNA can be detected at very low levels, indicating that Top2^K651A^ can, albeit with a very limited capacity, perform the SPR and therefore overall folding of the enzyme is not abolished. Y782F lacks the active site tyrosine and therefore cannot cut G-segment DNA [29]. It can bind to the G-segment and undergo rounds of ATP binding/hydrolysis as well as N-gate opening and closure. It is predicted to lack the ability to capture a T-segment due to space constraints within the N-terminal orifice of the enzyme in the absence of G-segment cleavage. G144I cannot bind nucleotide and therefore cannot lock the N-Gate closed [28]. For this reason it is unlikely to capture a T-segment and since T-segment capture stimulates G-segment cleavage, it has much reduced cleavage activity. *In vitro*, however, cleavage activity has been measured and given this event, the enzyme may sample conformations normally associated with T-transport even in the absence of ATP hydrolysis and T-segment capture. E66Q has 200-fold reduced ATP hydrolysis activity and therefore inefficiently performs conformation changes that promote T-transport, including DNA-Gate opening [26]. *In vitro* studies indicate that inefficient T-transport is followed by SPR arrest after release of the T-segment. A second SPR cycle is not possible because the N-Gate cannot open without release of the hydrolysis products. Top2-B44 is predicted to be defective in T-transport. The mutated residue is positioned where the TOPRIM-fold lies adjacent to the DNA binding domain. The mutant has a reduced rate of ATP hydrolysis (this study). L475A/L480P has wild type ATP hydrolysis activity but T-transport occurs at a much reduced rate [32]. G738D and P824S are positioned in the C-gate portion of the enzyme. These mutants have a much reduced rate of the SPR but are predicted to not affect the T-transport steps associated with DNA-gate opening, but rather would affect a later step of the SPR [31]. *Right column*, Indicates if checkpoint activation occurs upon expression of each mutant at endogenous levels in yeast cells depleted of Top2^deg^ during G1 (this study).

Type II topoisomerases make a transient double-strand break in one DNA helix (the Gate-segment), pass a second helix through the break (the Transported-segment), then re-ligate the G-segment (Figure 1) [1]–[3]. This Strand Passage Reaction (SPR) has been widely studied because it is the target of important classes of anti-microbial and anti-cancer drugs, as well as a large array of natural products [4], [5]. Upon chemical inhibition of Topo II, cellular checkpoint response pathways are activated that attempt to delay the cell cycle and thus prevent chromosome mis-segregation and/or cell death. Firstly, the DNA damage checkpoint response [4] is activated by a class of Topo II inhibitor, so-called Topo II “poisons”, that trap Topo II-DNA cleavage complexes. When locked in this conformation, the ternary DNA-protein-drug complex can deteriorate to produce DNA breaks that are recognized by the DNA damage checkpoint machinery. This cellular response has been extensively studied and its induction is not specific to DNA damage that results from Topo II poisons. A second class, referred to as Topo II catalytic inhibitors, including the bisdioxopiperazines, do not induce enzyme-mediated DNA cleavage, but block the overall catalytic activity of Topo II by trapping the enzyme in a state in which the N-terminal gate is closed (see Figure 1). These inhibitors activate alternative checkpoint controls that arrest the cell cycle [6]–[10], but the cell cycle control mechanisms that are employed are not well understood [6] and it is of particular interest to determine how checkpoint signaling occurs in the absence of DNA cleavage. A current subject of much controversy is whether the checkpoint detects dysfunctional Topo II directly or if cells utilize other well-characterized mechanisms, for example the spindle assembly checkpoint, to indirectly monitor Topo II activity via topological changes in chromosomal DNA.

Using the bisdioxopiperazine ICRF-193 to activate checkpoint signaling in human cells, one study has demonstrated binding of the DNA damage checkpoint signaling protein MDC1 to the C-terminus of Topo II, which thus induces cell cycle arrest in G2-phase [11]. Because the divergent C-terminal region is dispensable for the strand passage reaction of the enzyme, one explanation of these data is that inefficient or arrested catalysis is structurally transferred to the C-terminus where signaling complexes then assemble. However, the bisdioxopiperazines can bind to Topo II enzymes that are not associated with DNA [12] and are therefore not engaged in the strand passage reaction. Thus, it has not been determined whether checkpoint controls have the ability to directly assess the strand passage reaction.

A further complication is that a mitotic (as opposed to a G2-phase) checkpoint response is also activated by ICRF-193 in human cells and in yeast cells, Topo II (*top2*) mutants arrest mitotic progression *via* Mad2-dependent inhibition of anaphase onset [9], [13]. Mad2 is a spindle checkpoint protein that prevents chromatid disjunction when chromosomes are not properly bioriented on the mitotic spindle. The well-understood mechanisms that activate Mad2 can be initiated by aberrant tension at kinetochores [14], [15]. This indicates that Topo II (named Top2, in yeast) dysfunction in yeast mutants, or in mitotic human cells treated with ICRF-193, may be sensed indirectly, via structural changes at the centromere regions of chromosomes. This is an attractive hypothesis given that Topo II is concentrated at the centromere regions [16]–[18], that the strand passage reaction alters DNA topology [19], [20], and that some yeast *top2* mutant strains have altered centric chromatin structure [21]. It therefore remains unknown whether checkpoints that respond to Topo II inhibition involve novel signaling mechanisms or simply activate the already well-understood mitotic checkpoint pathways.

Here we demonstrate that, in yeast, the strand passage reaction of Topo II is directly monitored and that checkpoint signaling originates from the defective homodimeric enzyme at specific conformational states within the strand passage reaction. Checkpoint activation under these defined conditions requires the C-terminal region of the enzyme, supporting the hypothesis that a structural transference of defective catalysis to the enzymatically inert C-terminus allows checkpoint signaling. This is the first example of a checkpoint mechanism that directly monitors the catalytic cycle of a single enzyme.

## Results/Discussion

### Yeast *top2* Mutant Alleles Activate a Non-Conventional Mad2-Dependent Checkpoint

We previously studied yeast strains harboring the *top2-B44* hypomorphic allele of Topo II which induces a Mad2-dependent cell cycle delay in G2 (equivalent to the metaphase stage of mitosis in mammalian cells). This checkpoint was not activated due to the accumulation of DNA stand breaks [13]. Correspondingly, the DNA damage checkpoint was not activated and the major kinases involved in the DNA damage response (Rad53 and Mec1) were dispensable for G2 checkpoint activation in *top2-B44* cells [13]. In contrast, the spindle checkpoint protein Mad2 was essential for G2 checkpoint activation, suggesting that aberrant chromosome structure, especially kinetochore structure, may be defective under conditions of limited Topo II activity. To test this hypothesis we examined chromosome attachment to the mitotic spindle apparatus in *top2-B44* cells and surprisingly found no evidence in favor of aberrant attachment of kinetochores to the mitotic spindle [13]. Intriguingly, the checkpoint delay was at least partially independent of other effectors of the spindle checkpoint response; Pds1 and Bub3 [13].

To understand the mechanism of checkpoint activation in *top2-B44* cells, we first examined if reduced Topo II activity affected chromosome condensation. We employed an assay characterized previously in which chromosome compaction is monitored using marked chromosomal loci on the long arm of chromosome IV in yeast [22]. This analysis revealed that temporally concomitant with checkpoint activation in *top2-B44* cells, there was no defect in chromosome linear compaction (Figure S1). In fact, chromosome condensation occurred just as efficiently as in wild type cells.

### In the Absence of Top2, DNA Topology Defects Do Not Activate the Mad2-Dependent G2 Checkpoint

**Figure 2:**
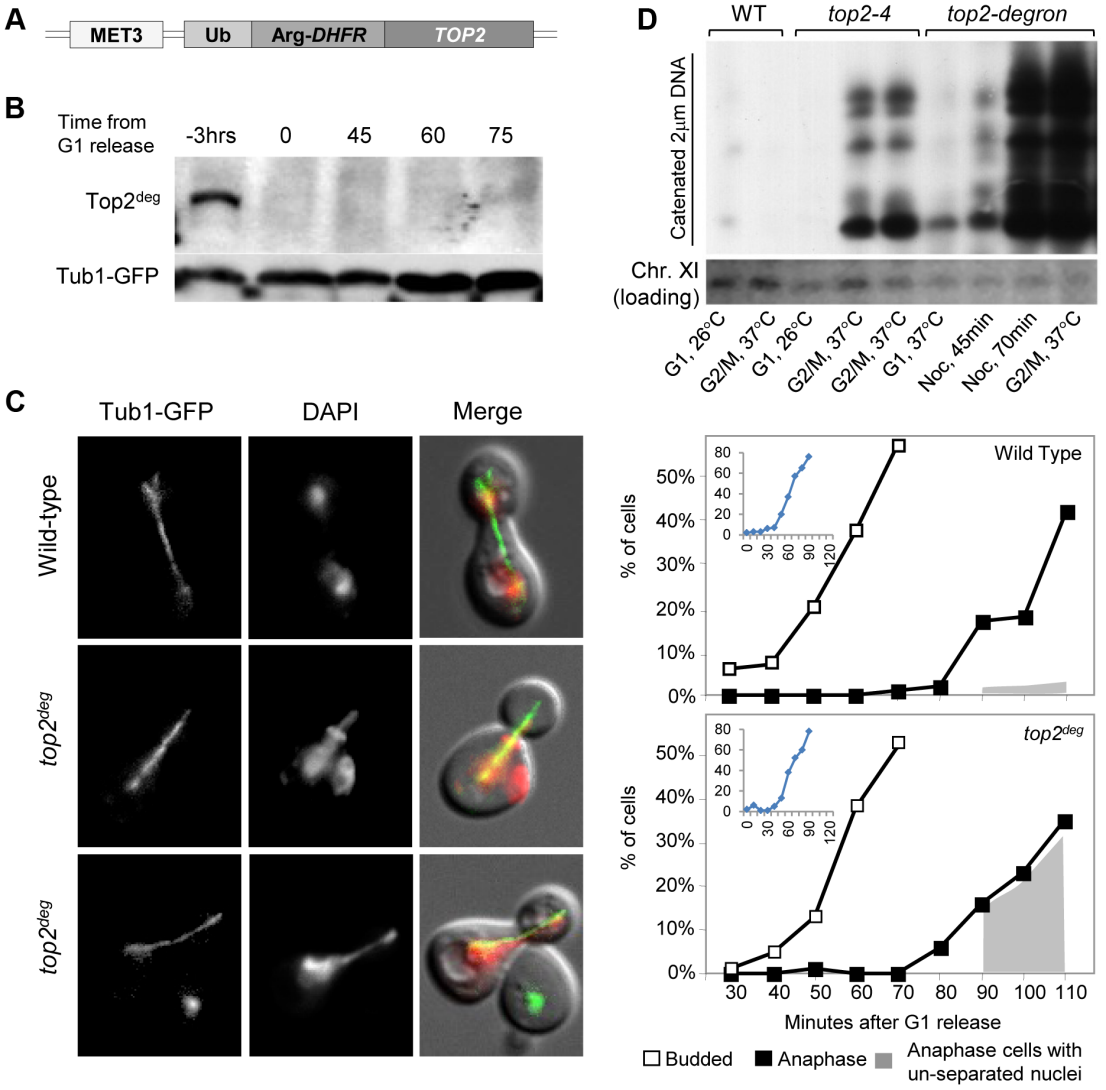
Characterization of a Yeast *top2* Degron Strain. **a**, Schematic of the chromosomal *MET3*-*top2* ^deg^ gene encoding thermo-labile Top2^deg^ protein and controlled transcriptionally *via* the presence or absence of methionine in the growth medium. **b**, Western blot of Top2^deg^. Temperature and carbon-source shifts (Figure S2) promote efficient degradation (Tub1-GFP, loading-control). **c**, Micrographs (left) and quantification (right) of failed nucleus segregation (DAPI) in anaphase cells with elongated spindles (Tub1-GFP) after Top2^deg^ was degraded in G1 and the subsequent cell cycle analyzed. Shaded region on graphs indicates the fraction of anaphase nuclei that were not segregated and the top left insets show expanded budding curves. **d**, CHEF gel analysis of separated chromosomes after Southern blotting to detect catenated topoisomers of the endogenous 2-micron plasmid. Wild type (WT), *top2-4* and *top2^deg^* strains were initially arrested in G1 (alpha-factor) at 26°C (37°C in the case of *top2^deg^*) or subsequently allowed 2 hours to reach G2/M at 37°C in the presence of nocodazole to prevent anaphase onset (G2/M, 37°C). For *top2^deg^*, additional samples were taken at 45 min and 70 min following alpha factor release at 37°C with nocodazole (Noc. 45 min, Noc. 70 min).

Because a wide variety of assays failed to provide any evidence that aberrant DNA topology or DNA breaks in *top2-B44* cells were the cause of G2 checkpoint activation (*i.e.* indirect consequences of perturbed Topo II activity), we next considered the possibility that cells may directly monitor the enzyme activity of Topo II. A system was established to control expression and degradation of Top2 in yeast using a degron allele (*top2* ^deg^) expressed from the *MET3* promoter (Figure 2a). Top2^deg^ contains a Ubiquitin-Arg-DHFR fusion which destabilizes folding at high temperature and becomes poly-ubiquitylated by the E3 ligase, Ubr1 [23]. Tight control of *top2* ^deg^ expression and Top2^deg^ degradation was achieved (Figure 2b and Figure S2). After G1 synchronization, Top2^deg^ was abolished to undetectable levels, allowing effects on progression through a subsequent cell cycle in the absence of Top2 to be observed, as previously described [24]. Under such conditions, if cells reached G2 unable to resolve DNA catenations arising during DNA replication, then upon anaphase entry these cells ought to fail to segregate their chromosomes. This was indeed the outcome in ∼90% of cells examined (Figure 2c), indicating that they reached G2 with extensive DNA topological defects arising under conditions of Top2-deficiency. To confirm this conclusion *via* biochemical means, we asked if *top2* ^deg^ cells grown under the above conditions (*i.e.* released from G1 after the depletion of Top2) reached G2 with catenated DNA. We isolated genomic DNA from such cells and following separation on CHEF gels, probed resulting Southern blots to detect the endogenous yeast circular 2-micron plasmid (as previously used to assess the catenation state of yeast genomic DNA; see Material and Methods). As a positive control we examined genomic DNA from *top2-4* cells grown in parallel under restrictive conditions. In both cases, extensively catenated 2-micron circular DNA was detected, whereas none was observed in samples from either mutant strain grown under permissive conditions or from wild type cells grown at high temperature (Figure 2d). Based on this molecular analysis of DNA catenation and the analysis of chromosome segregation *in vivo*, we conclude that following depletion of Top2^deg^ in G1, cells progress through the cell cycle with severely limited, if not absent, Top2 activity, resulting in persistent DNA hyper-catenation and failed chromosome segregation.

**Figure 3:**
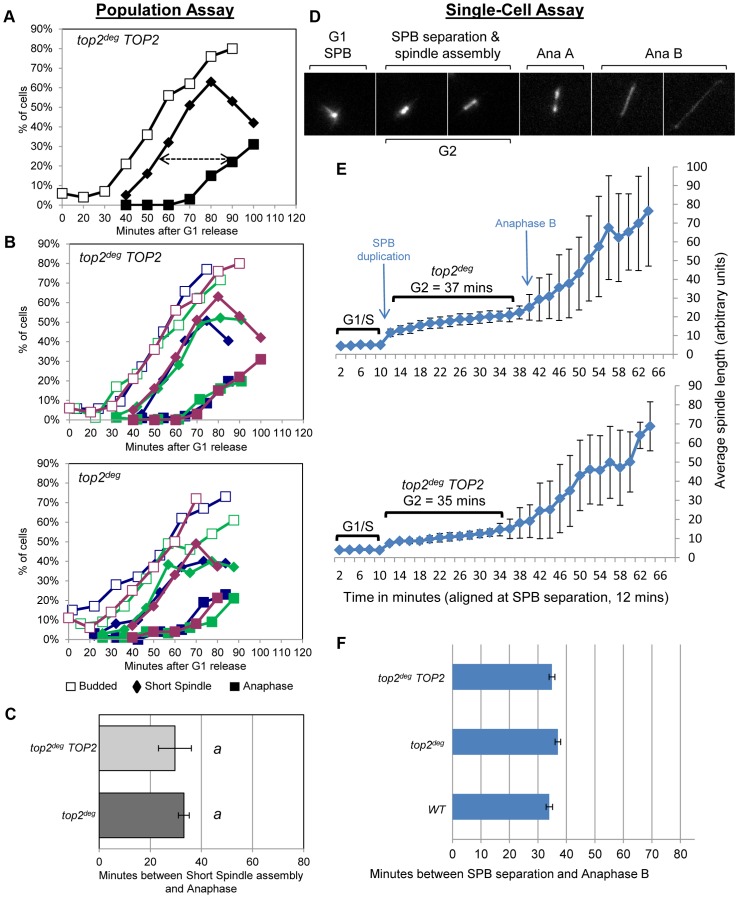
Analysis of G2/M Cell Cycle Checkpoint Activation in *top2* Mutants. **a–c**, Population Assays: kinetics of cell cycle progression based on budding (DIC microscopy) and spindle morphology (Tub1-GFP). Cell populations were returned to growth after depletion of Top2^deg^ and synchronization in G1 (alpha-factor). The interval between spindle assembly and anaphase (*a*, arrow) is not significantly different with wild-type endogenously expressed *TOP2* or after Top2^deg^ degradation in G1. Panel *b* shows overlaid repetitions of each experiment to assess variation (green/blue/purple, indicate three experimental repeats). Panel *c*, histogram plot of average G2/M duration; see Material and Methods for statistical analysis. **d–f**, Single-cell Assays: kinetics of spindle assembly and elongation in single cells based on digital time-lapse microscopy of strains expressing Tub1-GFP. Images in panel *d* show representative images of each morphological state (also see Movie S1). Panel *e*, plot of average spindle length (or SPB diameter; first five time points) versus time for single cells aligned on the x-axis at the time of SPB separation (*i.e.* at time point 12 min). Standard deviation of lengths shows that SPB diameter and G2 spindle length are relatively constant. Standard deviation of spindle length increases markedly as some cells enter anaphase. Panel *f*, plots average time interval between SPB separation and the initiation of spindle elongation in anaphase B (+/− s.e.).

The presence of DNA hyper-catenation must result in extensive chromosome topological defects, particularly at the centromere regions of chromosomes where Topo II is concentrated in mitosis. It can therefore be predicted that DNA hyper-catenation activates the spindle assembly checkpoint *via* aberrant centric (centromeric) chromatin. To test this hypothesis, we used two different assays to measure the approximate duration of G2 following depletion of Top2^deg^ in G1 and release of the cells from G1 synchrony. First we used a commonly used “population assay” where samples were taken from the population every 10–20 min and cells categorized into G1, G2 and anaphase morphologies based on mitotic spindle characteristics [24]. This yielded plots that revealed the approximate timing of anaphase spindle elongation relative to spindle assembly, which defines the duration of G2 (Figure 3a–c, Figure S3). Surprisingly, the length of G2 after Top2 depletion was very similar to that in the same strain but carrying an additional copy of wild type *TOP2* expressed from its endogenous promoter. Thus, the absence of Top2 did not induce a G2 cell cycle delay.

In a second assay, we studied cell cycle progression in single cells by digital time-lapse imaging (Figure 3d–f). Here, we recorded cells expressing GFP-tagged tubulin, acquiring z-stacks of images at a temporal resolution of 2 min. Except for growth in a micro-fluidic chamber, the experimental conditions of synchrony and Top2 depletion were identical to those used in the population assay. The resulting time-lapse movies were analyzed as follows to determine the average length of G2. First, we identified cells that completed the cell cycle within the duration of each movie. Second, we located the first time point (movie frame) in which two Spindle Pole Bodies (SPBs) could be observed (Figure 3d, second frame) and we then measured SPB diameter in the previous five movie frames to obtain a baseline SPB size. Next, we measured spindle length in all subsequent movie frames until full spindle elongation in anaphase had occurred. Approximate G2 length could then be determined by averaging the time interval from SPB separation to the onset of spindle elongation. We found that variation between cells was minimal as long as at least twenty cells were analyzed per strain. In addition to displaying simple histogram plots of G2 duration, we found that it was informative to plot average spindle length versus time, after the cells analyzed were aligned at the time of SPB separation (Figure 3e). In this manner, spindle lengths were largely very similar at each time point until a fraction of the cells initiated anaphase. Because spindle length differences are large in anaphase and are much larger than the length of the spindle in cells that were still in G2 at such time-points, standard deviation of spindle length became large, indicative of the trend towards anaphase onset among the cells recorded (Figure 3e, right side of the plots). In these single-cell assays, not unexpectedly, the kinetics of cell cycle progression was elongated due to growth within a static chamber, versus vigorous shaking used in the population assays. Nevertheless, these studies also revealed that the duration of G2 is very similar in cells depleted of Top2^deg^ versus cells producing Top2 at endogenous levels (Figure 3e,f).

We conclude that anaphase onset was not delayed when cells were allowed to progress from G1 after depletion of Top2 (Figure 3a–f, Figure S3). Presumably any centromeric chromatin defects that are a consequence of hyper-DNA catenation are not sufficient to activate a pre-anaphase checkpoint, including the spindle checkpoint. This conclusion is consistent with a previous study indicating that lack of Top2 in yeast does not delay mitotic progression [25] and studies in human cells in which checkpoint activation was not observed upon depletion of Topo II [10].

### Top2-B44 Activates a Mad2-Dependent G2 Delay when Associated with DNA

**Figure 4:**
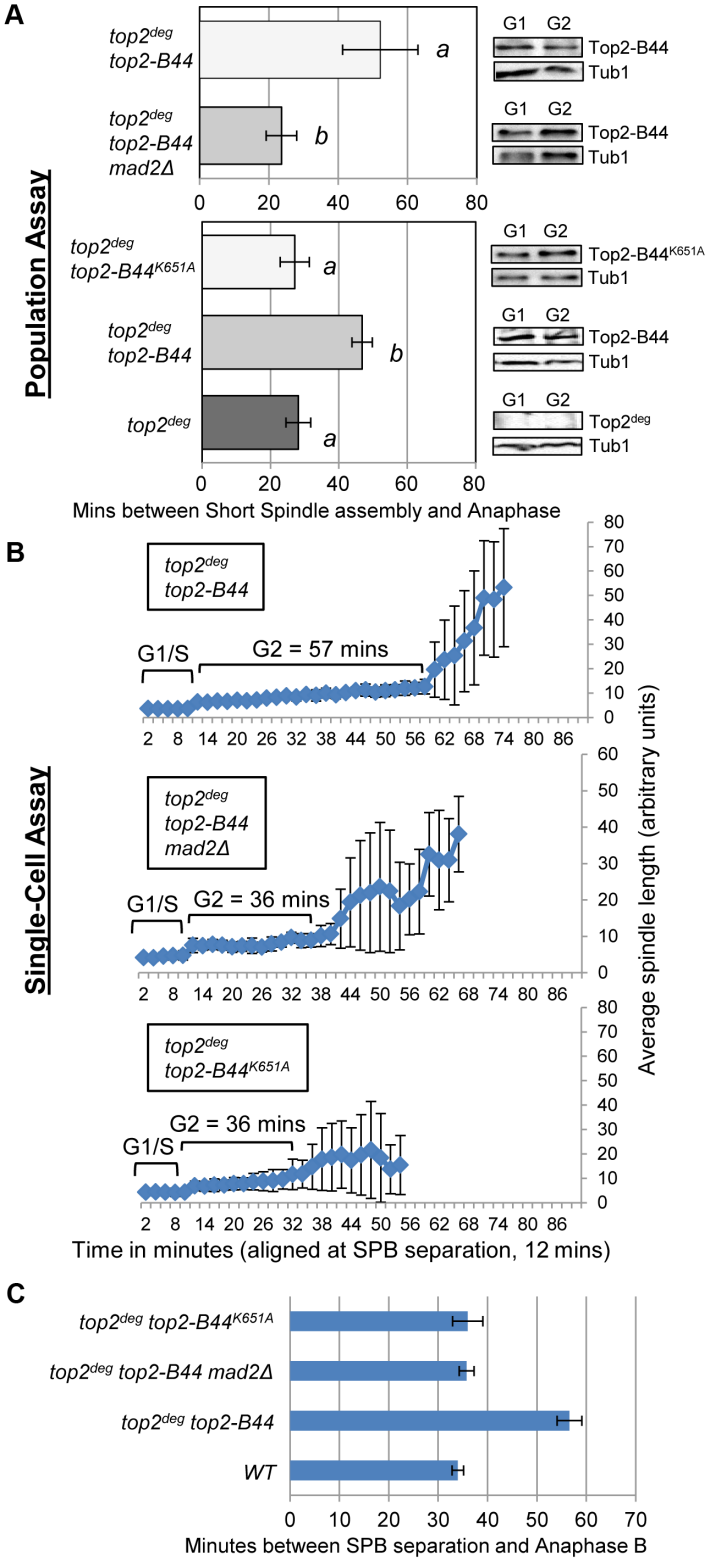
DNA-Associated Top2-B44 Activates a Mad2-Dependent G2/M Checkpoint. Analysis of the kinetics of cell cycle progression (see Figure 3) following depletion of Top2^deg^ and release from G1 synchrony in cells expressing endogenous levels of the indicated mutant Top2 proteins. **a**, Population Assays: Histogram plots show average G2/M duration; see Material and Methods for statistical analysis. Western blots show each Top2 mutant relative to Tub1 loading control at G1 and G2. *a* values are significantly different to *b* values in the histogram plots. Strains with the same letter are not significantly different. **b–c**, Single-cell assays: *b*, plots of average spindle length versus time for single cells aligned on the x-axis at the time of SPB separation (*i.e.* at time point 12 min). Error bars show standard deviation of lengths. *c*, Histogram plots of average time interval between SPB separation and the initiation of spindle elongation in anaphase B (+/− s.e.).

Because Top2 depletion did not activate checkpoint signaling but certain *top2* alleles are known to induce a Mad2-dependent cell cycle delay [13], we asked if a checkpoint response can be initiated in the *top2* ^deg^ strain after introduction of the *top2-B44* allele. When Top2^deg^ was depleted in G1 and Top2-B44 was expressed at endogenous levels (Figure 4a), population analysis revealed that anaphase initiation in the subsequent cell cycle was indeed delayed, indicating checkpoint activation (Figure 4a, Figure S3). Identical strains, but lacking the *MAD2* gene, did not delay before anaphase, confirming that the transient cell cycle arrest was Mad2 checkpoint-dependent. Single-cell assays confirmed these results (Figure 4b,c).

The above experiments demonstrated that hyper-catenated DNA was not sufficient for the induction of cell cycle arrest, but the presence of Top2-B44 protein did trigger checkpoint activation. A possible explanation is that cells can directly detect the aberrant activity of Top2-B44 and respond by initiating checkpoint signaling *via* Mad2. Similar to the effects of the drug ICRF-193, which can bind to Topo II in the absence of DNA [12], checkpoint activation in *top2-B44* cells could be due to the detection of abnormal Top2-B44 protein structure, independent of Top2 activity, or could be a result of direct monitoring of the SPR enzyme cycle. To distinguish between these alternatives, we took advantage of SPR mutants that have been characterized previously in structural/biochemical studies [26]–[32] (see Figure 1) and we performed both population and single-cell assays to confirm our findings. First, we asked whether Top2-B44 not only needs to be physically present in the cell, but also must be bound to DNA in order for checkpoint activation to occur. We introduced a K651A substitution into *top2-B44*. The conserved residue, K651, is present in a small, flexible linker region between the highly structured TOPRIM and WHD domains of Top2 that form the deep positively charged groove in which the G-segment DNA is housed [27]. Top2^K651A^ has a vastly reduced affinity for DNA [30] and cannot support cell viability (ref. 30 and data not shown). However, the K651A substitution is predicted not to destabilize the overall structure of Top2 [30]. Consistent with this prediction, Top2-B44^K651A^ was stable *in viv*o suggesting that it is correctly folded (Figure 4a). In the *top2* ^deg^ strain, Top2-B44^K651A^ was present at similar levels to Top2-B44, but did not induce a pre-anaphase delay (Figure 4a–c, Figure S3), indicating that the checkpoint is only activated when Top2-B44 is associated with DNA. A possible explanation is that the checkpoint monitors defective enzymatic cycles of Top2.

### Initiation of the SPR Is Required for Checkpoint Activation by Top2-B44

**Figure 5:**
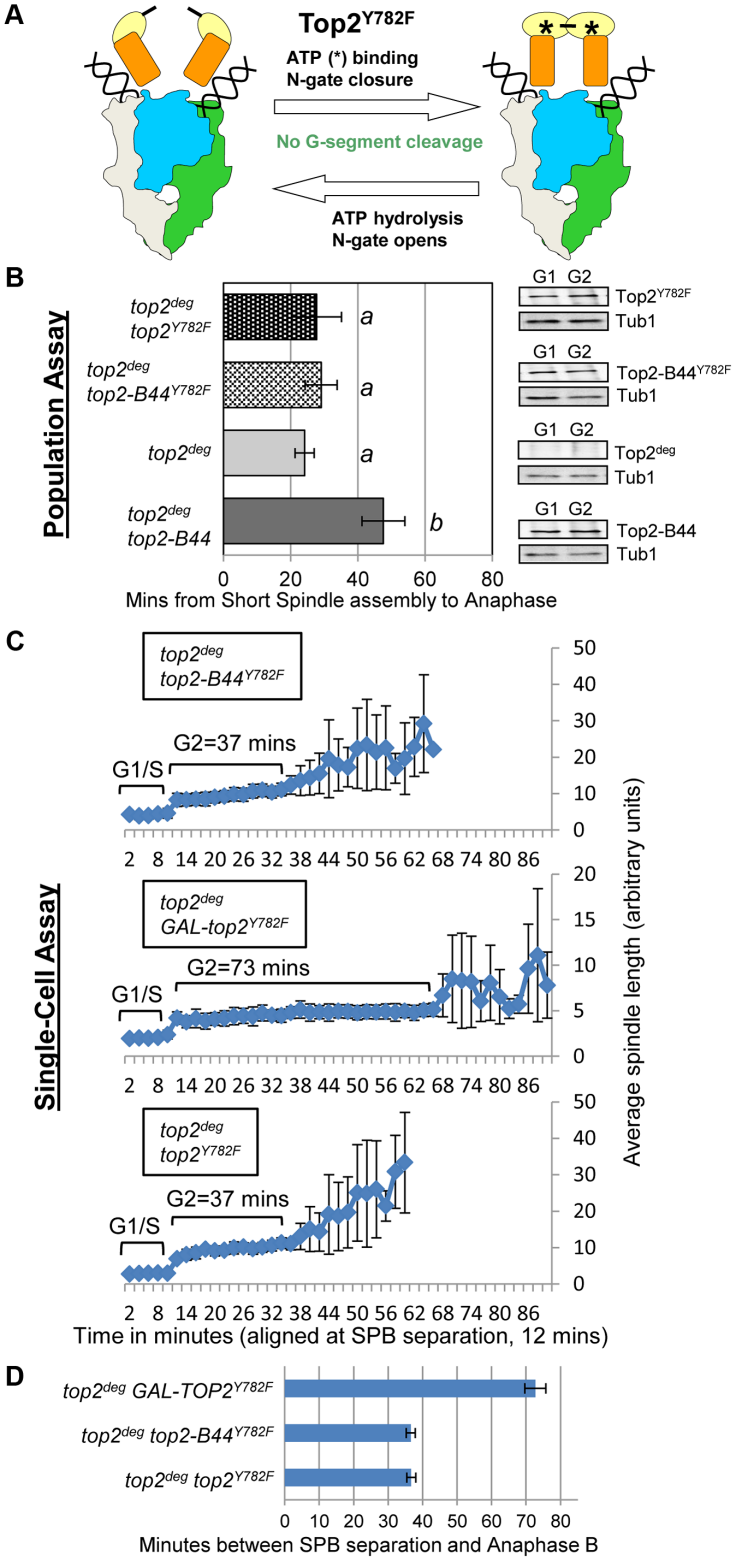
Checkpoint Activation *via* Top2-B44 Requires Initiation of the Strand Passage Reaction. **a**, Cartoon describing the catalytic defect in Top2^Y782F^ which cannot cleave G-segment DNA and thus performs non-productive cycles of ATP hydrolysis and N-gate closure/opening (see Figure 1 for complete Strand Passage Reaction and Key). **b–d**, Analysis of the kinetics of cell cycle progression (see Figure 3) following depletion of Top2^deg^ and release from G1 synchrony in cells expressing endogenous levels of the indicated mutant Top2 proteins. **b**, Population Assays: Histogram plots show average G2/M duration; see Material and Methods for statistical analysis. Western blots show each Top2 mutant relative to Tub1 loading control at G1 and G2. *a* values are significantly different to *b* values in the histogram plots. Strains with the same letter are not significantly different. **c–d**, Single-cell assays: *c*, plots of average spindle length versus time for single cells aligned on the x-axis at the time of SPB separation (*i.e.* at time point 12 min). Error bars show standard deviation of lengths. *d*, Histogram plots of average time interval between SPB separation and the initiation of spindle elongation in anaphase B (+/− s.e.).

We next asked if initiation of the SPR is required for checkpoint signaling to occur. The catalytic tyrosine of Top2 [29] was substituted with phenylalanine (Top2^Y782F^), resulting in an enzyme unable to cleave the G-segment DNA and capture a T-segment, though binding to the G-segment, binding of ATP and closure of the N-gate are not affected (Figure 1, Figure 5a). Top2^Y782F^ arrests bound to the G-segment but before the SPR can be initiated [29]. Population and single-cell analysis of *top2* ^deg^ cells expressing Top2^Y782F^ close to endogenous levels revealed a lack of checkpoint delay (Figure 5b–d, Figure S3). Because a previous study reported that over-expression of *TOP2^Y782F^* does activate a checkpoint response [25], we asked if this is the case in our strain background using a *GAL-TOP2^Y782F^* construct. Indeed, we did observe a G2 delay similar to the previous study, in agreement with the result that over-produced Top2^Y782F^ induces checkpoint activation (Figure 5c,d). We conclude that at least when present at endogenous levels, a Top2 enzyme that binds the G-segment but is trapped within a futile cycle of N-Gate opening and closure does not activate checkpoint signaling. We next substituted Y782 with phenylalanine in the Top2-B44 enzyme (Top2-B44^Y782F^). Similar to *top2* ^deg^ cells expressing Top2^Y782F^, this enzyme, expressed near endogenous levels, did not trigger checkpoint activation (Figure 5b–d, Figure S3). We infer that a defective step of the SPR of Top2-B44, subsequent to G-segment cleavage, is required for checkpoint activation, presumably because a downstream step of the SPR enzyme cycle is directly monitored.

### Defects in ATP Hydrolysis by Top2 Activate the Mad2-Dependent Checkpoint

**Figure 6:**
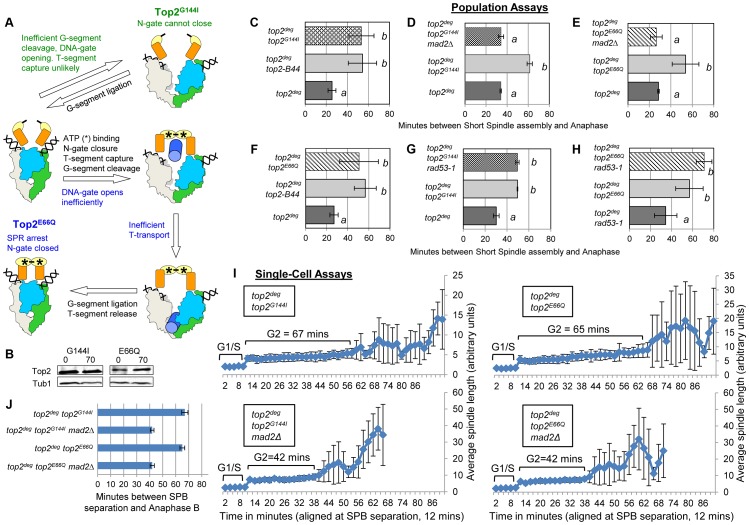
Defective Top2 ATP Binding and Hydrolysis Activate the Mad2-Depedent Checkpoint. **a**, Cartoon describing catalytic defects in Top2^G144I^ which cannot bind ATP (green annotations) and Top2^E66Q^ which cannot hydrolyze ATP (blue annotations). (See Figure 1 for complete Strand Passage Reaction and Key). **b–j**, Cell cycle analyses showing that Top2^G144I^ and Top2^E66Q^ activate Mad2-dependent but Rad53-independent checkpoint signaling. Analysis of the kinetics of cell cycle progression (see Figure 3) following depletion of Top2^deg^ and release from G1 synchrony in cells expressing endogenous levels of the indicated mutant Top2 proteins. (b–h) Population Assays: Histogram plots show average G2/M duration; see Material and Methods for statistical analysis. Western blots show each Top2 mutant relative to Tub1 loading control at G1 and G2. *a* values are significantly different to *b* values in the histogram plots. Strains with the same letter are not significantly different. (i,j) Single-cell assays: *i*, plots of average spindle length versus time for single cells aligned on the x-axis at the time of SPB separation (*i.e.* at time point 12 min). Error bars show standard deviation of lengths. *j*, Histogram plots of average time interval between SPB separation and the initiation of spindle elongation in anaphase B (+/− s.e.).

Top2^Y782F^ cannot cleave G-segment DNA and as a consequence it cannot adopt the closed-clamp form of the enzyme with a captured T-segment within the closed N-gate; there appears to be insufficient space within the N-terminal orifice of the dimeric enzyme when bounded by an intact G-segment [27]. To ask if T-segment capture and closure of the N-gate must occur for checkpoint activation, we analyzed *top2^G144I^*, a mutant that cannot bind ATP/ADP and thus cannot close the N-gate, but can, albeit inefficiently, cleave the G-segment (Figure 6a) [28]. Interestingly the checkpoint was activated by Top2^G144I^ similar to Top2-B44 (Figure 6b,c,i,j and Figure S4). Thus, N-gate closure with a captured T-segment is not required for checkpoint activation.

From biochemical and structural predictions, the Top2^G144I^ mutant ought not to produce frank DNA breaks and activate the DNA damage checkpoint. Indeed, Top2^G144I^ has reduced DNA cleavage activity because T-segment capture is inefficient [28]. Nevertheless, we asked if the activated checkpoint is Mad2 dependent, as is the case for Top2-B44 [13], or dependent on the DNA damage checkpoint kinase Rad53. Double mutants of *top2^G144I^* combined with a deletion of *MAD2* did not activate the checkpoint (Figure 6d,i,j and Figure S5), whereas double mutants combined with the checkpoint defective *rad53-1* allele or a *rad53*-null allele (*rad53Δ*) retained checkpoint signaling (Figure 6g, Figure S5 and Figure S9). Consistent with these findings, we failed to detect Rad53 phosphorylation in cells expressing Top2^G144I^ (Figure S9). Therefore, like *top2-B44*, the *top2^G144I^* cells arrest the cell cycle due to the activation of Mad2, and likely do not activate the DNA damage checkpoint. Consistent with these findings for Top2^G144I^ which cannot bind ATP, we observed that Top2^E66Q^, defective in ATP hydrolysis (Figure 6a) [26], activates the checkpoint in a Mad2-dependent, but Rad53-independent manner (Figure 6b,f,e,h–j, Figure S6 and Figure S9).

### A Defect in T-Segment Transit Activates the Mad2-Dependent Checkpoint Independently of Slow ATP Hydrolysis

**Figure 7:**
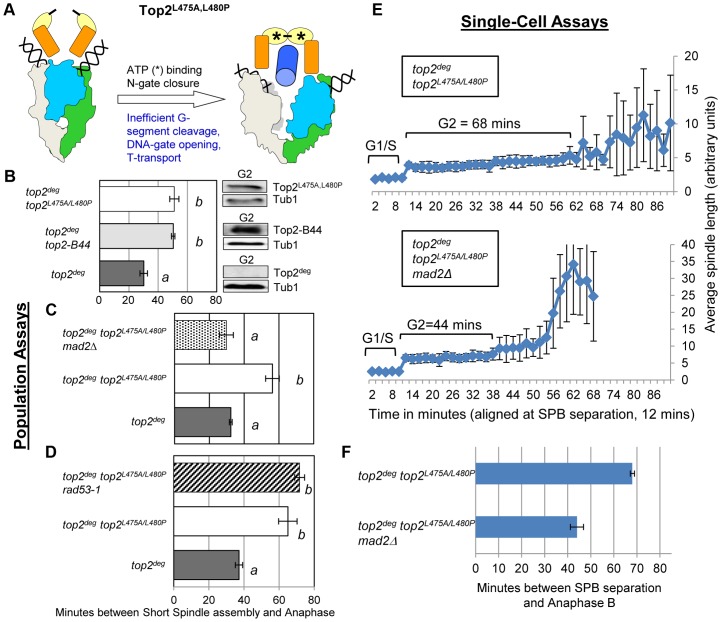
Defective T-Segment Transit may Activate the Mad2-Dependent Checkpoint. Cell cycle analyses showing that Top2^L475A,L480P^ activates Mad2-dependent but Rad53-independent checkpoint signaling. **a**, Cartoon describing catalytic defect in Top2^L475A,L480P^ which has a reduced rate of T-segment transport due to inefficient G-segment cleavage and DNA-gate opening. **b–d**, Population Assays: Histogram plots show average G2/M duration; see Material and Methods for statistical analysis. Western blots show each Top2 mutant relative to Tub1 loading control at G1 and G2. *a* values are significantly different to *b* values in the histogram plots. Strains with the same letter are not significantly different. **e–f**, Single-cell assays: *e*, plots of average spindle length versus time for single cells aligned on the x-axis at the time of SPB separation (*i.e.* at time point 12 min). Error bars show standard deviation of lengths. *f*, Histogram plots of average time interval between SPB separation and the initiation of spindle elongation in anaphase B (+/− s.e.).

Top2^E66Q^ and Top2^G144I^ have in common the inability to utilize the energy of ATP hydrolysis and consequently both enzymes transit very slowly through the coupled mechanism that drives the T-segment transport step of the SPR (Figure 1 and Figure 6a) [26], [28]. To ask if perturbed ATP hydrolysis *per se*, or conformational changes in the enzyme that promote T-segment transport, are monitored by the checkpoint machinery, we examined strains harboring *top2^L475A/L480P^*, which encodes an enzyme proficient in ATP hydrolysis, but defective in T-segment transport (Figure 7a) [32]. Strikingly this mutant activated the checkpoint to the same degree as the ATP binding and hydrolysis mutants, again in a Mad2-dependent, Rad53-independent manner, suggesting that the checkpoint is activated by inefficient T-segment transport (Figure 7b–f, Figure S7 and Figure S9).

**Figure 8:**
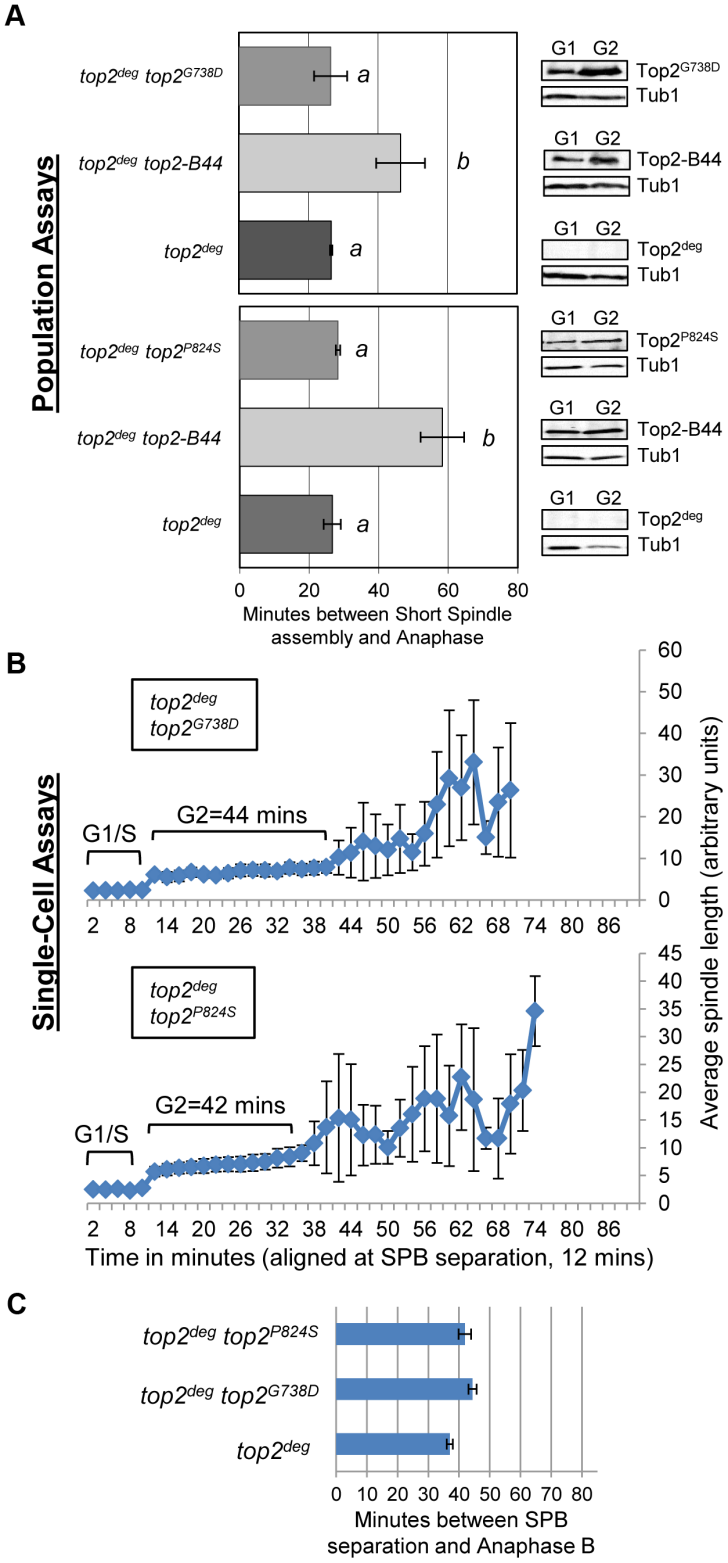
Slow Strand Passage does not Activate the Mad2-Dependent Checkpoint in the Absence of a T-Segment Transit Defect. Analysis of cell cycle kinetics in *top2* ^deg^ strains expressing Top2^G738D^ and Top2^P824S^ which have overall reduced rates of the catalytic cycle do not activate checkpoint signaling. **a**, Population Assays: Histogram plots show average G2/M duration; see Material and Methods for statistical analysis. Western blots show each Top2 mutant relative to Tub1 loading control at G1 and G2. *a* values are significantly different to *b* values in the histogram plots. Strains with the same letter are not significantly different. **b–c**, Single-cell assays: *b*, plots of average spindle length versus time for single cells aligned on the x-axis at the time of SPB separation (*i.e.* at time point 12 min). Error bars show standard deviation of lengths. *c*, Histogram plots of average time interval between SPB separation and the initiation of spindle elongation in anaphase B (+/− s.e.).

We then compared these data with other *top2* mutants in which the overall rate of the SPR is slow, but that are predicted not to be defective in T-segment transport through the DNA-gate: Top2^P824S^ and Top2^G738D^ (Figure 1) [31]. Neither mutant led to checkpoint activation (Figure 8a–c, Figure S8). Based on the analysis of these mutants, checkpoint activation does not require: (1) DNA strand breaks, (2) a defect in N-gate opening or closure, or (3) a defect in ATP binding or hydrolysis. Furthermore, an overall reduced rate of strand passage is not sufficient for checkpoint activation. The mutants that activate the checkpoint have in common a defect in the T-segment transport step of the SPR, which is associated with specific conformational states of the enzyme (Figure S10).

### Top2-B44 Hydrolyzes ATP Slowly

**Figure 9:**
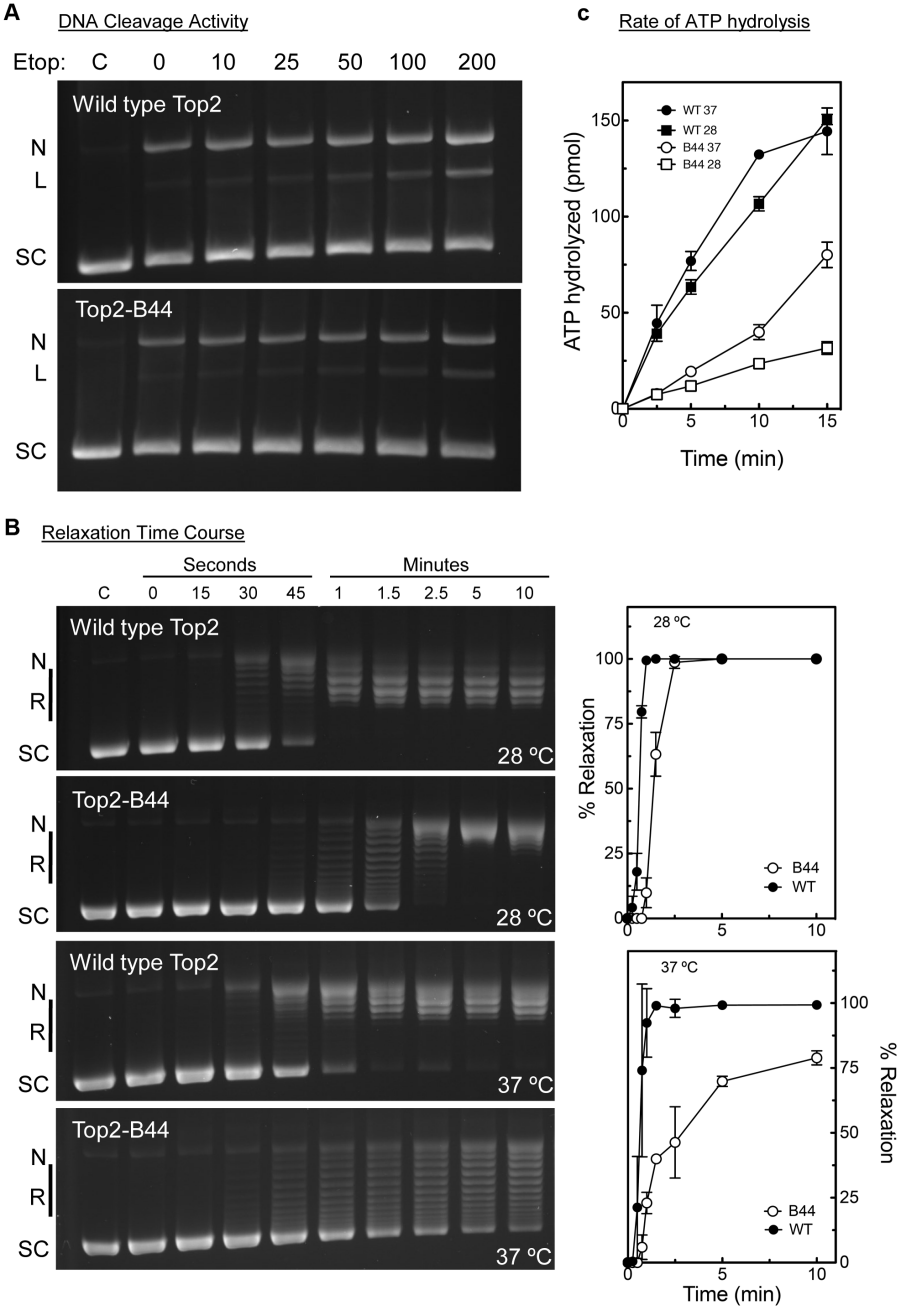
Top2-B44 is Defective in ATP Hydrolysis. **a**, Cleavage activity of Top2-B44. Agarose gel electrophoresis of supercoiled (SC) plasmid DNA either untreated (C) or after incubation with purified wild type Top2 or Top2-B44 enzymes in the presence of increasing concentrations of etoposide (Etop). N = nicked forms, L = linear form. The linear form indicates plasmid that was cut by Top2 and re-ligation blocked by binding of etoposide to the Top2-DNA complex. **b**, Relaxation activity of Top2-B44. *Left panel*, Agarose gel electrophoresis of supercoiled (SC) plasmid DNA either untreated (C) or after 0–15 min. incubated with purified wild type Top2 or Top2-B44 enzymes. L = Linear form, R = relaxed topoisomers. *Right panel*, Quantification of relaxation activity of purified wild type (WT) Top2 or Top2-B44 (B44) enzymes versus time at 37°C and 28°C. **c**, Rate of ATP hydrolysis by wild type (WT) Top2 and Top2-B44 (B44) enzymes at 37°C and 28°C (measured by the release of free phosphate).

Top2-B44 had not been characterized biochemically, but based on our analysis of the other mutants, we would predict that Top2-B44 is defective in the T-transport step of the SPR. To address this hypothesis, we purified wild type and Top2-B44 enzymes to homogeneity from yeast (see Material and Methods). First, we asked if Top2-B44 has an altered propensity to cleave supercoiled DNA in the presence of the Topo II poison etoposide. This assay can determine whether the enzyme has a propensity to stall with a cut G-segment. This cleavage activity assay (Figure 9a) revealed that Top2-B44 does not display increased DNA breakage in the presence of etoposide, which is consistent with our failure to detect DNA damage in *top2-B44* cells [13] (Figure S9) and is consistent with Mad2-dependent checkpoint activation rather than Rad53-dependent checkpoint activation. We then performed relaxation activity time course experiments to determine the rate of relaxation of supercoiled plasmid DNA (Figure 9b). This analysis demonstrated that Top2-B44 relaxes supercoiled DNA more slowly (∼2.5 to 3-fold) than wild type Top2 at 37°C. Most interestingly, however, Top2-B44 had a reduced rate of ATP hydrolysis (Figure 9c), consistent with checkpoint activation resulting from a defect in the T-transport step of the SPR, and consistent with the biochemical defects of Top2^G144I^, Top2^E66Q^ and Top2^L475A/L480P^ that all activated a Mad2-dependent G2 checkpoint.

### The C-Terminus of Top2-B44 Is Required for Mad2-Dependent Checkpoint Activation

**Figure 10:**
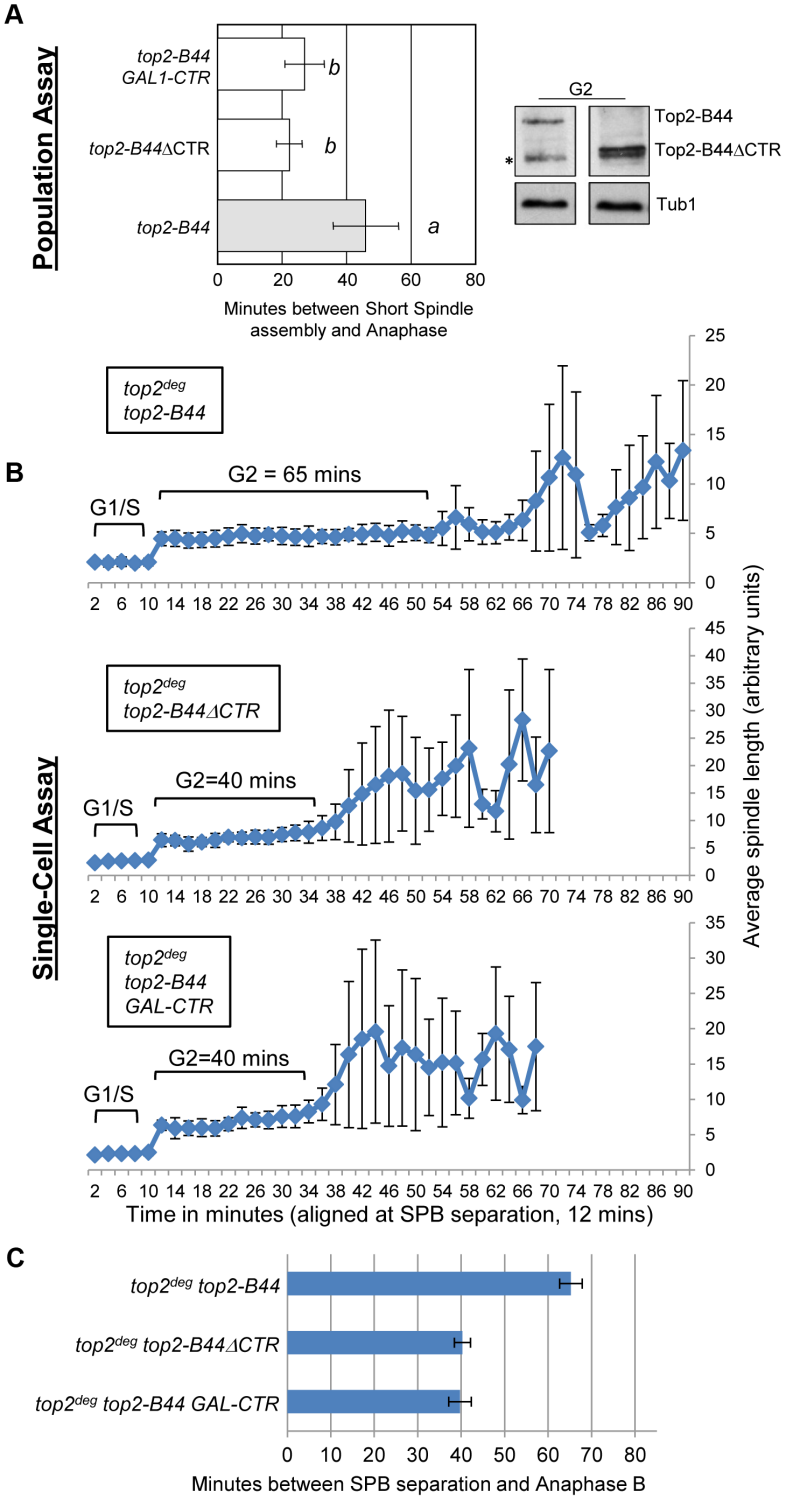
The C-Terminal Region (CTR) is Required for Checkpoint Activation by Top2-B44. Top2-B44^ΔCTR^ does not activate checkpoint signaling and over-production of the CTR fragment of Top2 abrogates checkpoint signaling in *top2-B44* cells. **a**, Population Assays: Histogram plots show average G2/M duration; see Material and Methods for statistical analysis. Western blots show each Top2 mutant relative to Tub1 control at G1 and G2 (in part d, * indicates a non-specific band). *a* values are significantly different to *b* values. Strains with the same letter are not significantly different. **b–c**, Single-cell assays: *b*, plots of average spindle length versus time for single cells aligned on the x-axis at the time of SPB separation (*i.e.* at time point 12 min). Error bars show standard deviation of lengths. *c*, Histogram plots of average time interval between SPB separation and the initiation of spindle elongation in anaphase B (+/− s.e.).

Topo II undergoes major conformation changes as part of the SPR, particularly during the step of the enzyme cycle where the T-segment is transported through the holoenzyme [27], [28]. Our data indicate that a specific conformation might be recognized by the checkpoint machinery. In this case, a determinant may exist within the Topo II quaternary structure that participates in checkpoint signaling. This hypothesis stems from the discovery that, in human cells, the checkpoint signaling protein MDC1 binds to the C-terminus of Topo II [11]. We therefore tested the hypothesis that specific *top2* mutants activate a Mad2-dependent checkpoint response *via* the catalytically inert C-terminal region (CTR). Because the CTR (residues 1321 to the C-terminus) is dispensable for the SPR and for cell viability in yeast [33], we could construct strains expressing either wild type *TOP2* with a truncated CTR (*top2* ^ΔCTR^) or *top2-B44* with a truncated CTR (*top2-B44* ^ΔCTR^). Strikingly, we observed that Top2-B44^ΔCTR^ does not activate checkpoint signaling, demonstrating that the CTR is required for Mad2 activation (Figure 10a–c).

The C-terminal regions of eukaryotic Topo II enzymes are divergent, and thus it is not clear whether the CTRs of human and yeast Topo II possess a conserved binding site for checkpoint proteins. Nevertheless, if checkpoint signaling complexes are recruited to the CTR of yeast Top2, then over-production of the CTR in isolation may sequester the relevant factor(s) and perhaps abrogate checkpoint signaling. This was indeed the outcome when the CTR was expressed from the strongly inducible *GAL1* promoter in the *top2-B44* strain (Figure 10a–c). These experiments therefore provide evidence that specific defects in the SPR transduce to the C-terminal region, resulting in the activation of checkpoint signaling. This could occur *via* the physical interaction of checkpoint proteins with the CTR, although other explanations of the data remain, such as over-produced CTR having dominant effects on chromatin that interfere with checkpoint signaling.

### Top2-B44 Induces G2 Cell Cycle Delay Independently of Ndc10 and without Gross Mad2 Recruitment to Kinetochores

**Figure 11:**
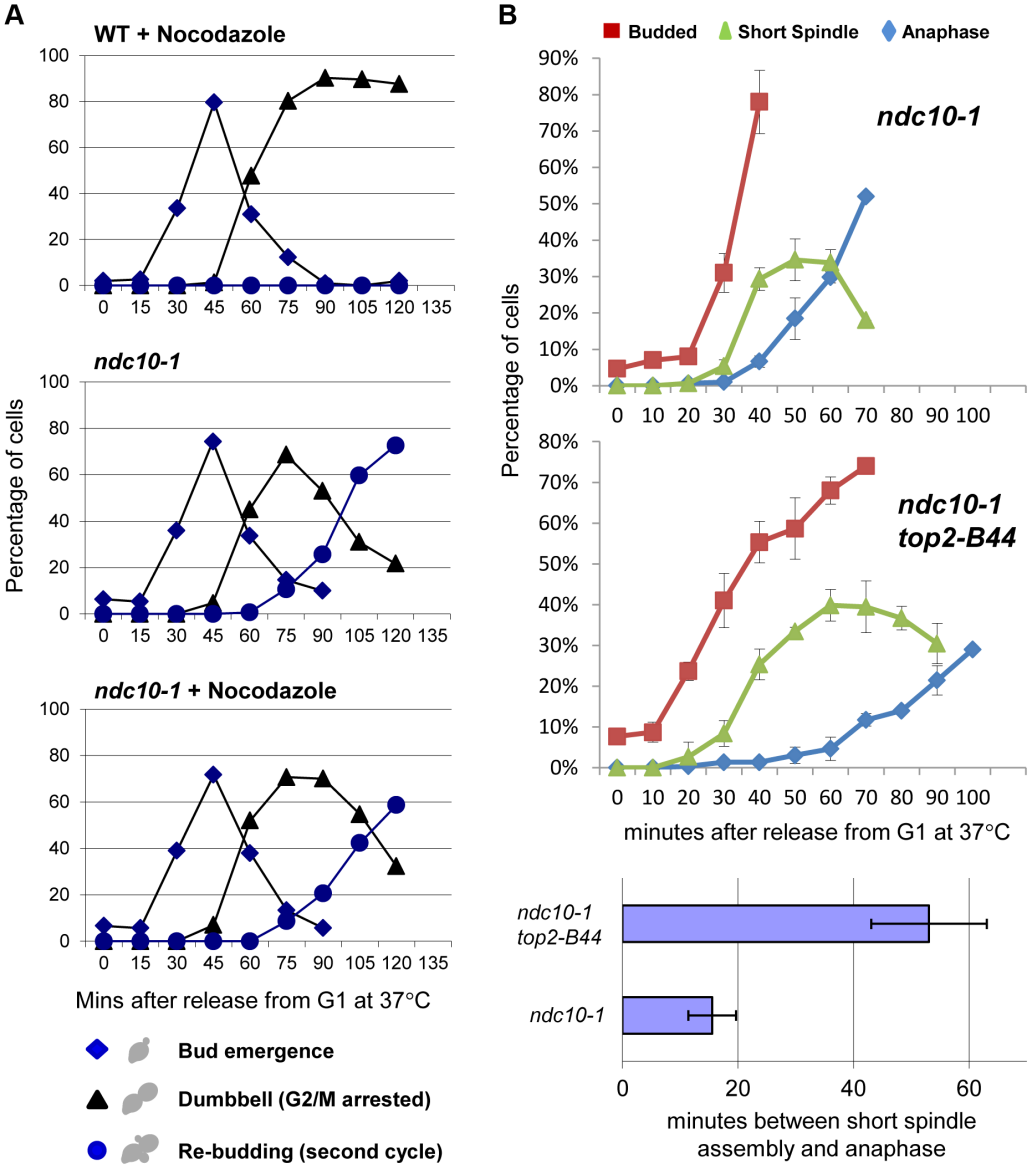
Checkpoint Activation by Top2-B44 is Independent of Kinetochores. **a–b**, Top2-B44 induces checkpoint activation in G2 under conditions where the kinetochore protein Ndc10 was inactivated in the preceding G1-phase. *a*, Analysis of re-budding (progression into a second cell cycle) with or without nocodazole after release from G1 synchrony (alpha factor). During synchrony, the strains were shifted to the restrictive temperature for the *ndc10-1* allele, thus destroying kinetochores in G1-phase, as previously described [34]. *b*, Population analysis of the kinetics of cell cycle progression based on budding (DIC microscopy) and spindle morphology (Tub1-GFP) in the indicated strains after release from G1 synchrony. During synchrony, the strains were shifted to the restrictive temperature for the *ndc10-1* allele, exactly as in panel *a*. Error bars on the line graphs show standard deviation between three repeats of the experiment. Histogram plot: The interval between spindle assembly and anaphase based on three repetitions of the experiment; see Material and Methods for statistical analysis.

Dissecting the role of the CTR and the molecular basis of Mad2 activation, remain important future goals. We previously found that checkpoint activation in *top2-B44* cells does not coincide with a defect in chromosome biorientation, and that some spindle checkpoint proteins (including Bub3 and the spindle checkpoint target, Pds1) are dispensable for the observed G2 delay [13]. Because these data suggested that a non-conventional spindle checkpoint is activated in *top2-B44* cells, we asked if the G2 delay requires the kinetochore protein Ndc10. In the absence of Ndc10, although kinetochores lose their structural integrity, cells are able to progress through the cell cycle and complete mitosis [34]. However, since Mad2 activation must occur at kinetochores when the spindle checkpoint is triggered, *ndc10* mutant cells cannot arrest prior to anaphase in the presence of microtubule poisons such as nocodazole [34]. We confirmed these data in our strain background in order to determine conditions that efficiently inactivate the temperature sensitive Ndc10-1 protein in conjunction with cell cycle synchrony in G1-phase. Upon release into the cell cycle, such cells failed to arrest in the presence of nocodazole, progressing into a second cell cycle almost as quickly as untreated cells (Figure 11a). Strikingly, however, under identical conditions but in the absence of nocodazole, *top2-B44 ndc10-1* cells delayed in G2-phase (Figure 11b). Therefore, checkpoint activation in *top2-B44* cells is independent of Ndc10.

**Figure 12:**
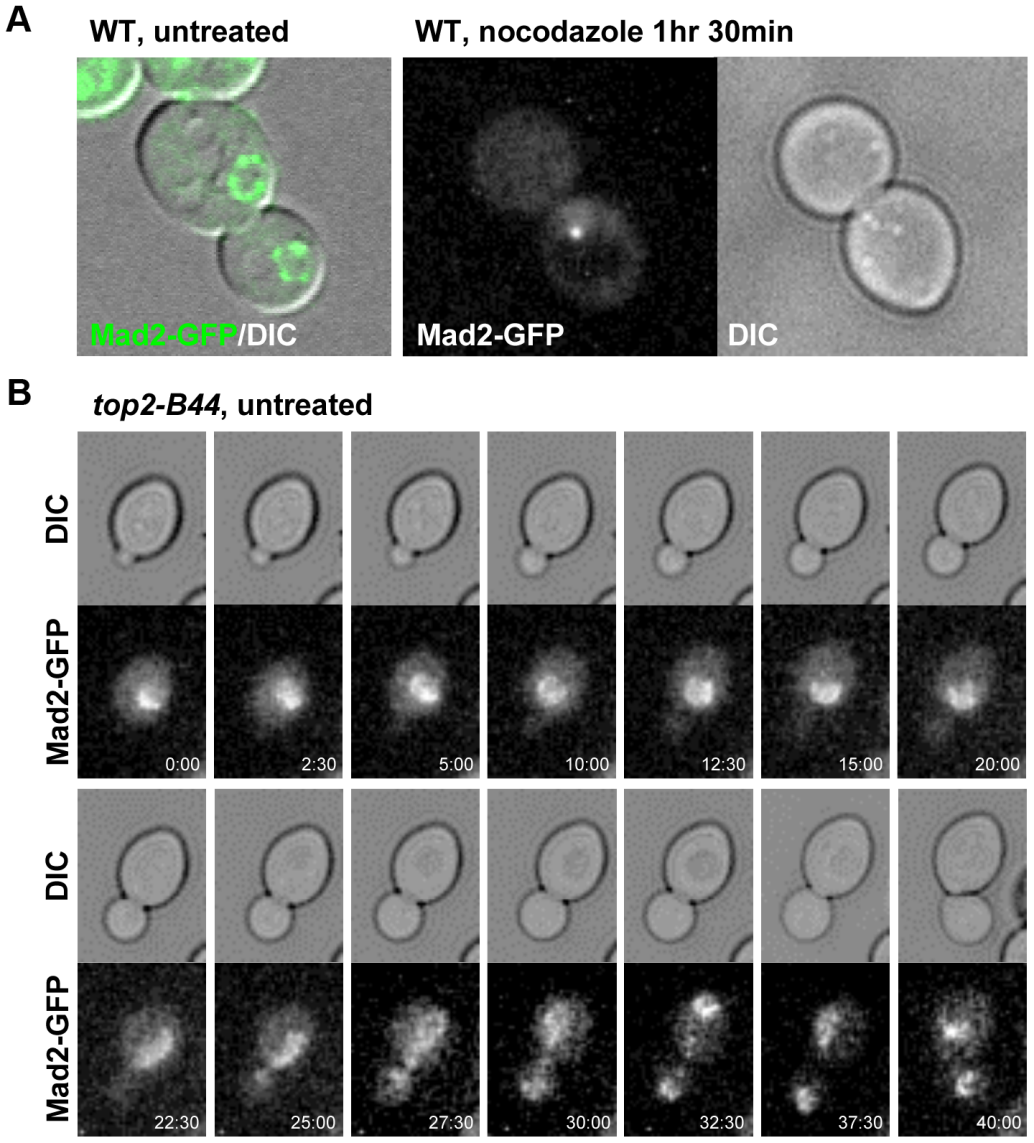
Mad2-GFP does not Re-Localize to Kinetochores in *top2-B44* Cells. **a**, Expression of Mad2 tagged with three tandem repeats of GFP localizes either diffusely or at the periphery of the nucleus in untreated wild type cells (left panel). Upon treatment with nocodazole Mad2-GFP re-localizes to a single discrete focus (right panel). **b**, Time-lapse analysis of Mad2-GFP from bud emergence (min 0:00) to cytokinesis (min 40:00) in untreated *top2-B44* cells.

The above data led to the prediction that Mad2 becomes activated in *top2-B44* cells independently of kinetochores. We therefore asked if Mad2 is recruited to kinetochores upon checkpoint activation in *top2-B44* cells. First we expressed Mad2 tagged with three tandem GFP epitopes in wild type cells in order to verify that we could observe re-localization of Mad2 upon activation of the spindle checkpoint. Indeed, as previously reported [35], within 90 minutes of nocodazole treatment, most cells possessed a single discrete focus of Mad2-GFP (Figure 12a, right panel, and data not shown). In the absence of nocodazole, we did not observe discrete foci of Mad2-GFP in either wild type or *top2-B44* cells. Rather, Mad2-GFP was either diffusely localized or localized to structures at the periphery of the nucleus (Figure 12a, left panel, Figure 12b and data not shown), consistent with previous studies showing that Mad2 localizes to nuclear pores in the absence of spindle checkpoint activation [35]. To ensure that Mad2-GFP does not localize to discrete foci in *top2-B44* cells, we filmed such cells by time-lapse microscopy at 2.5 minute intervals to observe complete cell cycles (Figure 12b, Movie S2). Analysis of more than 50 cells failed to reveal any Mad2-GFP foci in *top2-B44* cells. We conclude that Mad2 does not re-localize to kinetochores and that kinetochores are dispensable for checkpoint activation in *top2-B44* cells. These findings are consistent with the genetic analyses described above, and suggest a novel mechanism of Mad2-dependent checkpoint activation *via* Top2-B44.

### Concluding Remarks

It will be important to extend the studies presented here to improve our understanding of mammalian mitotic checkpoint controls. It has been known for some time that inhibition of Topo II in human cells induces a G2 checkpoint delay that is dependent on the DNA damage checkpoint kinase ATM [36]. The checkpoint signaling pathway activated in G2 human cells is therefore clearly distinct from the Mad2-dependent checkpoint that becomes activated in yeast *top2* mutants. This likely is the case because the yeast cell cycle is organized quite differently from human cells. Since budding yeast cells assembly their mitotic spindles during S-phase and achieve chromosome biorientation (equivalent to mammalian metaphase chromosome alignment) before the completion of DNA synthesis, they do not possess a G2 cell cycle phase that temporally precedes mitosis. As a result there is no true cell cycle transition between G2 and mitosis and thus no opportunity for biochemical regulation at an equivalent stage to the mammalian G2-prophase transition. Perhaps for this reason, budding yeasts seem to rely heavily on regulation of anaphase onset *via* Mad2 and other mechanisms that control stability and activity of anaphase inhibitors. Nevertheless, an equivalent cell cycle control to the one we have described in yeast does appear to function in human cells. When metaphase human cells are treated with the Topo II inhibitor ICRF-193, a transient cell cycle delay prior to anaphase onset is observed that endures for approximately 45–60 minutes before anaphase is attempted and cells exit mitosis [37]. Little is known about this cellular response, however, and it will be intriguing to determine how similar it may be to the yeast checkpoint. In addition, it should be noted that much higher doses of ICRF-193 have been reported to silence the spindle checkpoint under conditions where cohesin proteins have been depleted, which results in defective tension at kinetochores and thus activates the spindle checkpoint [38]. The mechanistic basis of this phenomenon remains poorly understood and it is not clear how different doses of ICRF-193 can have very different consequences *in vivo*. However, it is interesting to note that parallels can be drawn between the yeast Top2^E66Q^ mutant and a wild-type enzyme in the presence of ICRF-193, since in both cases ATP can bind to the topoisomerase but cannot be hydrolyzed. The Top2^E66Q^ mutant, which activates the Mad2-dependent checkpoint in yeast, therefore phenocopies a situation where each Topo II holoenzyme is bound to ICRF-193, without there being an excess of the drug. It is clear that a comprehensive understanding of the interplay between mitotic checkpoints and Topo II function will provide important information in terms of the consequences of inhibiting the enzyme in the context of anti-microbial and anti-cancer treatments.

In this study, we have presented evidence that a Topo II responsive checkpoint is activated through a novel mechanism that detects the enzyme cycle rather than a DNA lesion. DNA topology defects, including hyper-catenation, are not sufficient to trigger checkpoint signaling. Rather, the evidence supports a mechanism whereby the checkpoint machinery directly monitors the SPR of Topo II to ensure that sister chromatid separation can occur in anaphase. The data also indicate that the checkpoint is distinct from other checkpoints. Such a mechanism of checkpoint control could have evolved due to the abundance of naturally occurring Topo II inhibitors such as plant secondary metabolites that interrupt the enzyme cycle [4], [5]. The SPR mutants described here may mimic some of the effects of these inhibitors in that they accumulate specific SPR intermediates that may form a structural platform for assembly of a signaling complex. We propose that checkpoint sensor proteins bind to Topo II when blocked or delayed at specific steps in the SPR. If the assembly of signaling complexes at the CTR of Topo II is a conserved mechanism of checkpoint activation across eukaryotes, then the nature of interaction with the Topo II CTR must have diverged since no homolog of the human MDC1 checkpoint protein exists in yeast. Further understanding of such Topo II-checkpoint protein interactions could be clinically valuable because current efforts aim to identify drugs that inhibit Topo II without activating the checkpoint controls that are protective to tumors cells.

## Material and Methods

### Yeast Strains and Plasmids

The yeast strains used in this study are haploid derivatives of BF264-15 15DU (see Table S2). Yeast strains were modified according to standard yeast genetic approaches [39]. Plasmids were mutagenized by site-directed mutagenesis according to Liu and Naismith [40].

### Growth Conditions to Inactivate Top2^deg^

Step 1: cells were grown to an OD of 2.0–6.0 overnight at 26°C in synthetic raffinose medium lacking methionine and tryptophan (Figure S2). Step 2: for synchrony, cells were diluted to OD 0.2 in rich raffinose medium at 26°C with α factor (concentration varying from 1∶2200 to 1∶3500 of a 1 mg/ml stock). After 1 h, galactose was added to a final concentration of 4% (Step 3). Following another 30 min growth the temperature was raised to 35°C (Step 4). After a further 30 min growth (2 hours total with α factor) α factor was washed off with water that was pre-warmed to 35°C. The cells were released into YPG at 35°C (Step 5).

### Determination of G2/M Period

#### Population Assays

G2/M period was determined as previously described [13], [24]. Briefly, at each time point and for each strain, at least 100 cells were scored for spindle and budding morphology. The G2/M cell cycle interval was determined by plotting cell cycle categories against time. G2/M was defined as the period after 20% of cells had formed a G2 spindle until 20% of cells formed an anaphase spindle. Each experimental strain was analyzed a minimum of three times. The data in Figure S3, S4, S5, S7, S7, S8 are aligned relative to bud emergence since this signals cell cycle entry and is the variable parameter of cell cycle progression following release from G1 synchrony [13]. For statistical analysis, values for G2/M from the yeast cell cycle analyses were analyzed using One-Way Anova, using the Tukey HSD post-hoc analysis (significance p = 0.05). For values see Table S1.

#### Single-cell Assays

Following Top2^deg^ deletion and G1 synchrony (as performed for the population assays), strains were grown in a microfluidic chamber housed on a DeltaVision microscope system with defined environmental conditions (humidified at 34°C). Z-stack images were captured at 2 min. intervals. ImageJ (Fiji) was used to measure spindle lengths and average spindle length (+/− s.d.) versus time was plotted with each cell aligned on the x-axis at the first time point in which spindle pole separation could be observed. For histogram plots of the average duration of G2, the interval between spindle pole body separation and anaphase onset was calculated (+/− s.e.). Between 20 and 40 single cells were analyzed for each strain.

### Microscopy

Spindle morphologies were visualized using *TUB1-GFP* [41] as described previously [24]. Nuclear morphologies were visualized using a method from Juan Martinez (Purdue University). Cells were grown under the conditions specified previously and were collected (500 µl) by centrifugation. The resulting pellet was washed once with 1 ml 1× PBS, resuspended in 1.4 ml of filter-sterilized 4% p-formaldehyde solution (4% p-formaldehyde, 3.5% sucrose in water), and incubated for 20 min at room temperature. Cells were then centrifuged at 3000 *g* and the pellet was washed with 1 ml wash buffer (1.2 M sorbitol, 100 mM potassium phosphate, pH 7.5). Cells were resuspended in 1 ml 1× PBS containing 5 µl of 1 mg/ml DAPI solution and incubated at room temperature for 3 min in the dark. Cells were washed once with 1× PBS and resuspended in the remaining 1× PBS after decanting. Anaphase cells were identified based on spindle morphology and were scored for separated or un-separated nuclei. During a normal mitosis, anaphase cells go through an intermediate stage where the nuclei are stretched, but have not separated completely (this accounts for the low percentage of anaphase cells in wild-type with un-separated nuclei).

### Western Blots

Proteins were extracted by collecting 15 mls of cells and resuspending in 1 mL 0.25 M NaOH and 1% BME solution. Resuspensions were placed on ice for 10 min. 160 µl of 50% TCA was added, the solution inverted, and placed on ice for 10 min. The extracts were then pelleted at 14,000 *g* for 10 min at 4°C. Supernatant was decanted and the pellet resuspended in 1 mL of ice-cold acetone. Extracts were pelleted again and supernatant decanted. The extract was dried for 3 min at 55°C. The dried extract was resuspended in 100 µl of 2× protein-loading buffer and neutralized with 5 µl of 1 M Tris base. Western blots were performed using the following antibodies: 1∶1000 dilution of anti-Top2 (TopoGen), 1∶6000 dilution of anti-Flag (Pierce) and a 1∶2500 dilution of anti-GFP (Clontech). Secondary antibody, HRP-conjugated goat anti-rabbit (Pierce) was used at 1∶5000 and HRP-conjugated goat anti-mouse IgG (Invitrogen) was used at 1∶5000.

### CHEF Gels and Southern Blotting

Preparation of yeast cell agarose plugs for CHEF gel electrophoresis was performed as preciously described [42] and were run on a CHEF DRIII electrophoresis system (Bio-Rad) at 11°C, 60–120 sec switch for 18 hours at 6 V/cm with 120° angle. Southern blots were probed to detect catenated 2-micron circle DNA as previously described [43].

### Purification and Biochemical Analysis of Top2-B44

Wild type Top2 and Top2-B44 were purified after over-expression in yeast as previously described [44]. Top2 reactions were carried out as described [44] using supercoiled pBR322 to monitor ATP-dependent relaxation and cleavage activity. ATPase assays were performed as described by Osheroff et al. [45]. Reaction mixtures contained 45 nM yeast topoisomerase II, 5 nM negatively supercoiled pBR322 DNA, and 1 mM [γ-32P] ATP in a total of 40 µl of relaxation buffer. Mixtures were incubated at 28°C and 37°C, and 2 µl samples were removed at time intervals up to 15 min and spotted on polyethyleneimine-impregnated thin layer cellulose chromatography plates (EMD Chemicals). Plates were developed by chromatography in freshly made 400 mM NH_4_HCO_3_ and analyzed using a Bio-Rad molecular imager FX. ATP hydrolysis was monitored by the release of free phosphate.

## Supporting Information

Figure S1 *top2-B44* Does Not Have a Chromosome Condensation Defect.
(PDF)

Figure S2 Conditional Expression and Degradation of Top2^deg^.
(PDF)

Figure S3 Cell Cycle Population Analyses of SPR Mutants: *top2-B44, top2^Y782F^, top2-B44^K651A^*.
(PDF)

Figure S4 Cell Cycle Population Analyses of SPR Mutants: *top2-B44, top2* ^G144I^.
(PDF)

Figure S5 Cell Cycle Population Analyses of SPR Mutants: *top2-B44, top2* ^G144I^.
(PDF)

Figure S6 Cell Cycle Population Analyses of SPR Mutants: *top2-B44, top2* ^E66Q^.
(PDF)

Figure S7 Cell Cycle Population Analyses of SPR Mutants: *top2-B44, top2* ^L475A/L480P^.
(PDF)

Figure S8 Cell Cycle Population Analyses of SPR Mutants: *top2-B44, top2* ^G738D^, *top2* ^P824S^.
(PDF)

Figure S9 Rad53 is Dispensable for Checkpoint Activation in *top2* Strand Passage Mutants.
(PDF)

Figure S10 SPR Defects That Activate Checkpoint Signaling.
(PDF)

Movie S1 Yeast strain expressing Tub1-GFP filmed during growth in a micro-fluidic chamber at controlled temperature, captured using a DeltaVision microscope system.
(MOV)

Movie S2 A *top2-B44* yeast strain expressing Mad2-3×GFP filmed during growth in a micro-fluidic chamber at controlled temperature, captured using a DeltaVision microscope system.
(AVI)

Table S1 Values for G2/M duration for each strain analyzed using One-Way Anova.
(PDF)

Table S2 Yeast strains used in this study.
(PDF)
