# Supplementary material for: Direct Monitoring of the Strand Passage Reaction of DNA Topoisomerase II Triggers Checkpoint Activation
Source: PLoS Genet. 2013 Oct 3;9(10):e1003832. doi: 10.1371/journal.pgen.1003832 (PMC3789831; doi:10.1371/journal.pgen.1003832)
Supplement: Figure S1 — top2-B44 Does Not Have a Chromosome Condensation Defect. (PDF) [file pgen.1003832.s001.pdf]

Top2-B44 does not have a chromosome condensation defect

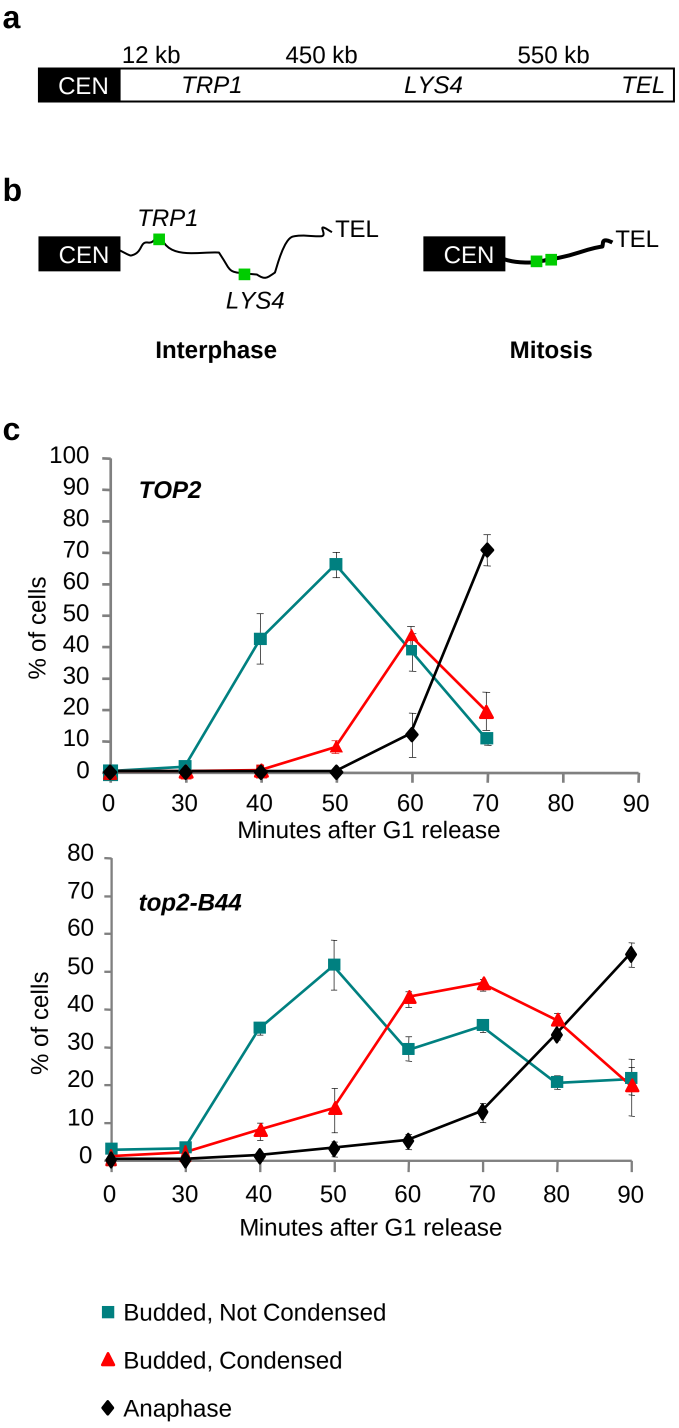

Furniss et al. Supplemental Figure 1

Figure S1

***top2-B44* does not have a Chromosome Condensation Defect**

a-b, Schematic description of the chromosome condensation assay used, based on Vas *et al.* 2007 [22]. LacO tandem repeats integrated at two loci (*TRP1* and *LYS4*) on the right arm of chromosome IV are visualized by expression of GFP-LacI. In interphase, the loci are visualized in live cells as separated foci (green squares), whereas in mitosis the foci coalesce upon chromosome condensation. **c**, Cell cycle analysis of chromosome condensation in wild type and *top2-B44* strains. Cells were arrested in G1 using mating pheromone and analyzed by microscopy over one subsequent cell cycle at 10 minute intervals. At each time point, the % of cells in each of the indicated categories was determined. Budding (blue line) is approximately coincident with the initiation of DNA replication. Chromosome condensation (red line) is consistent with the timing of mitotic entry relative to bud emergence. Anaphase (black line) in wild type cells occurs with normal timing, 10-15 minutes after chromosome condensation. In *top2-B44* cells, anaphase was delayed due to checkpoint activation. Chromosome condensation occurs in *top2-B44* cells with similar timing as the *TOP2* cells.
