# Supplementary material for: Direct Monitoring of the Strand Passage Reaction of DNA Topoisomerase II Triggers Checkpoint Activation
Source: PLoS Genet. 2013 Oct 3;9(10):e1003832. doi: 10.1371/journal.pgen.1003832 (PMC3789831; doi:10.1371/journal.pgen.1003832)
Supplement: Figure S2 — Conditional Expression and Degradation of Top2deg. (PDF) [file pgen.1003832.s002.pdf]

Conditional Expression and Degradation of Top2^{deg}

Steps to Inactivate Degron	Conditions	Top2-degron transcription	Gal-Ubr1 transcription	Protein folding
1	Overnight culture SR-Met 26ºC	ON	OFF	Stable
2	YPR 26ºC + αF	OFF	OFF	Stable
3	4% Galactose added to culture	OFF	ON	Stable
4	Culture shifted to 35ºC	OFF	ON	Unstable
5	Wash and release into YPG at 35ºC	OFF	ON	Unstable

Furniss et al. Supplemental Figure 2

Figure S2

Conditional Expression and Degradation of Top2^{deg}

From top to bottom, media and temperature combinations are described that allow efficient depletion of Top2^{deg} protein from yeast cells concurrent with synchrony in G1 phase of the cell cycle.
