# Supplementary material for: Direct Monitoring of the Strand Passage Reaction of DNA Topoisomerase II Triggers Checkpoint Activation
Source: PLoS Genet. 2013 Oct 3;9(10):e1003832. doi: 10.1371/journal.pgen.1003832 (PMC3789831; doi:10.1371/journal.pgen.1003832)
Supplement: Figure S4 — Cell Cycle Population Analyses of SPR Mutants: top2-B44, top2 G144I. (PDF) [file pgen.1003832.s004.pdf]

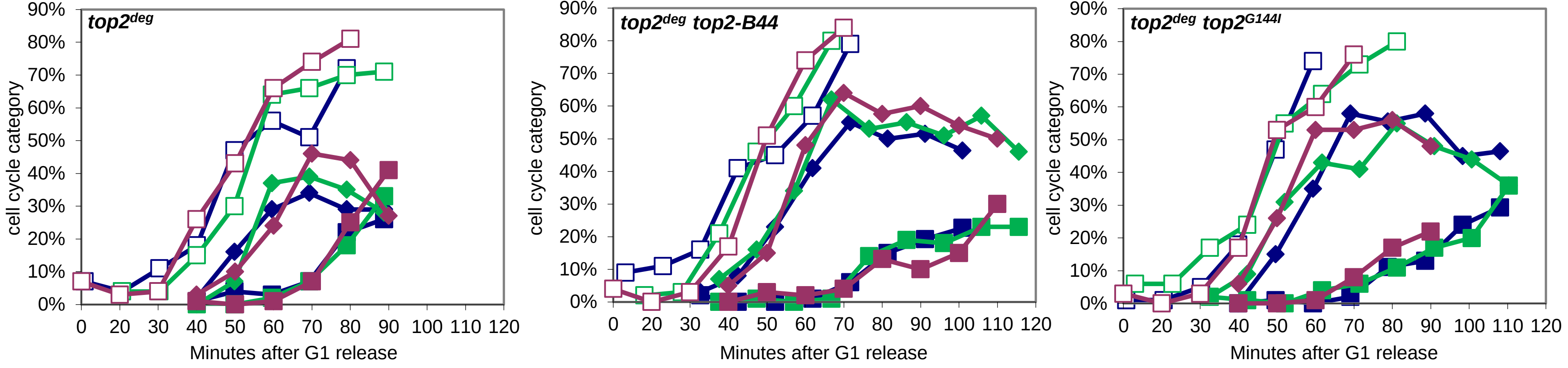

Furniss et al. Supplemental Figure 4

Figure S4

Cell Cycle Population Analyses of SPR Mutants: *top2-B44, top2*^{G144I}

Each yeast strain was synchronized in G1 and Top2^{deg} depleted as described in the Material and Methods section. Upon release, samples were taken at 10 minute intervals to score the % cells that had undergone bud emergence (open squares), G2 spindle assembly (diamonds) and anaphase (closed squares). Each analysis was performed a minimum of three times (purple, blue and green plots) and the data aligned based on bud emergence. The G2/M interval (time period between spindle assembly and anaphase) was calculated for each strain as described in the Material and Methods section.
