# Supplementary material for: Direct Monitoring of the Strand Passage Reaction of DNA Topoisomerase II Triggers Checkpoint Activation
Source: PLoS Genet. 2013 Oct 3;9(10):e1003832. doi: 10.1371/journal.pgen.1003832 (PMC3789831; doi:10.1371/journal.pgen.1003832)
Supplement: Figure S9 — Rad53 is Dispensable for Checkpoint Activation in top2 Strand Passage Mutants. (PDF) [file pgen.1003832.s009.pdf]

Rad53 is dispensable for checkpoint activation in *top2* strand passage mutants

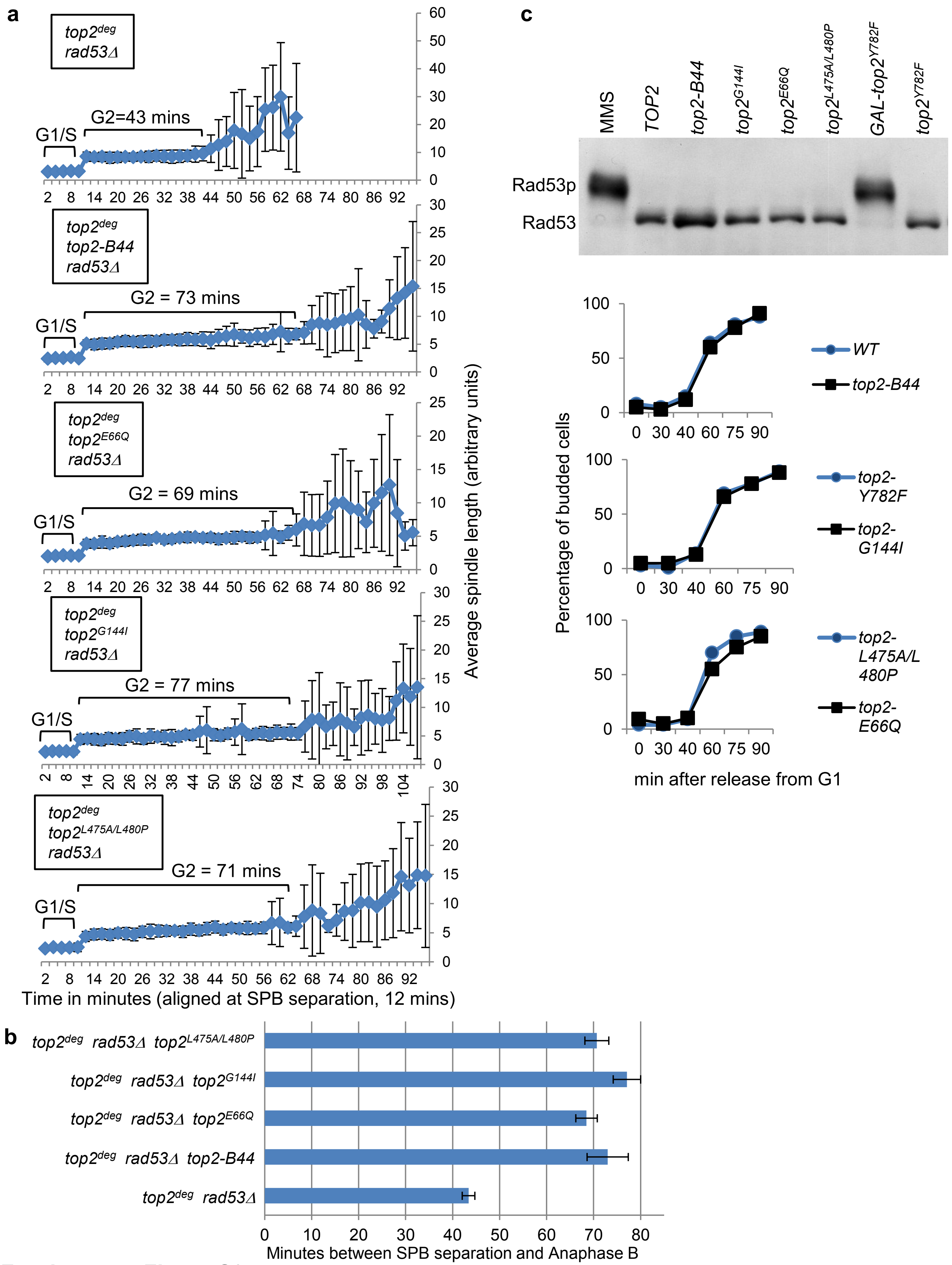

Furniss et al. Figure S9

Figure S9

Rad53 is Dispensable for Checkpoint Activation in *top2* Strand Passage Mutants

a-b, Analysis of the kinetics of cell cycle progression (see Figure 3) following depletion of Top2^{deg} and release from G1 synchrony in cells expressing endogenous levels of the indicated mutant Top2 proteins. *a*, Single-cell assays: plots of average spindle length versus time for single cells aligned on the x-axis at the time of SPB separation (*i.e.* at time point 12min). Error bars show standard deviation of lengths. *b*, Histogram plots of average time interval between SPB separation and the initiation of spindle elongation in anaphase B (+/- s.e.) derived from the data in panel *a*. **c**, Analysis of Rad53 phosphorylation (electrophoretic band shift assay) in *top2* mutants. Following depletion of Top2^{deg} and release from G1 synchrony in cells expressing endogenous levels of the indicated mutant Top2 proteins and harboring a Flag-tagged endogenous Rad53 allele, cell cycle progression was monitored in the presence of nocodazole (to prevent DNA breakage at cytokinesis) by counting the % of budded cells (histogram plots). Samples were taken for Western blot analysis of Flag-Rad53 (top panel) at time point 90 min. As positive controls for Rad53 phosphorylation, cells were either treated with 0.05% methyl methanesulfonate (MMS) for 2 hours or were induced to over-produce Top2^{Y782F} for 3 hours (*GAL-TOP2*^{Y782F}).
