# Supplementary material for: Direct Monitoring of the Strand Passage Reaction of DNA Topoisomerase II Triggers Checkpoint Activation
Source: PLoS Genet. 2013 Oct 3;9(10):e1003832. doi: 10.1371/journal.pgen.1003832 (PMC3789831; doi:10.1371/journal.pgen.1003832)
Supplement: Figure S10 — SPR Defects That Activate Checkpoint Signaling. (PDF) [file pgen.1003832.s010.pdf]

SPR defects triggering checkpoint signaling

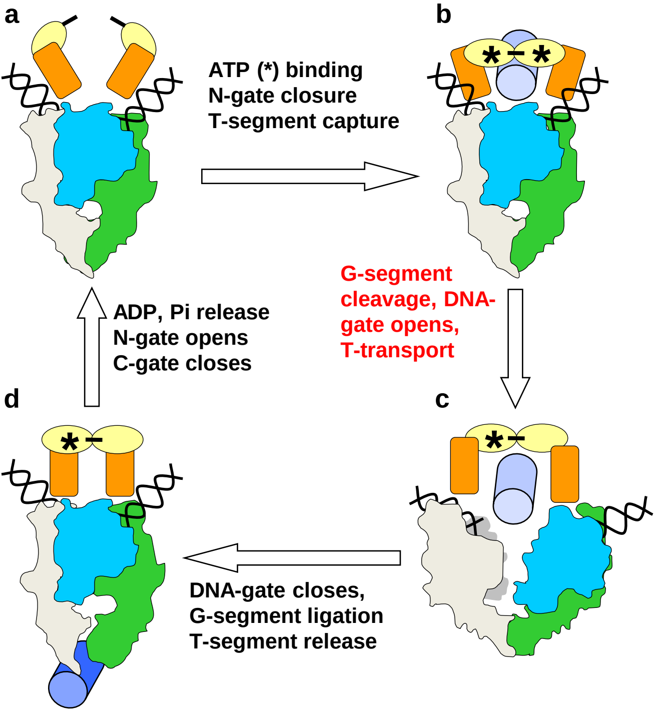

Furniss et al. Supplemental Figure 10

Figure S10

SPR Defects That Activate Checkpoint Signaling

Comparison of the SPR of *top2* mutants that activate the checkpoint indicates that a defect in DNA transport results in checkpoint signaling. **a**, Bound G-segment, open N-gate. **b**, Captured T-segment, closed N-gate. This form is not predicted to occur in the absence of G-segment cleavage. **c,** Open DNA-gate, T-segment transit in progress. **d**, Closed DNA-gate and G-segment re-ligation. Key: refer to Figure 1.
