# Supplementary material for: Direct Monitoring of the Strand Passage Reaction of DNA Topoisomerase II Triggers Checkpoint Activation
Source: PLoS Genet. 2013 Oct 3;9(10):e1003832. doi: 10.1371/journal.pgen.1003832 (PMC3789831; doi:10.1371/journal.pgen.1003832)
Supplement: Table S1 — Values for G2/M duration for each strain analyzed using One-Way Anova. (PDF) [file pgen.1003832.s013.pdf]

Table S1

Values for G2/M duration (see Material and Methods section) for each strain analyzed using One-Way Anova. The Tukey HSD post-hoc analysis (significance $p = 0.05$) indicates which strains had significantly different G2/M lengths. The left column indicates the Figure in which each data set is graphed and compared. The second column indicates the initial strain, while the third column is the query strain. p-values are in the last column, significance was set at $p \leq 0.05$.

Figure	Strain 1	Strain 2	Significance
3c	*top2*^{deg} *TOP2*	*top2*^{deg}	.423
4a	*top2*^{deg} *top2-B44*	*top2*^{deg} *top2-B44 mad2Δ*	.022
4a	*top2*^{deg} *top2-B44*	*top2*^{deg}	.002
		top2^{deg} *top2-B44*^{K651A}	.001
4a	*top2*^{deg}	*top2*^{deg} *top2-B44*	.002
		top2^{deg} *top2-B44*^{K651A}	.932
4a	*top2*^{deg} *top2-B44*^{K651A}	*top2*^{deg} *top2-B44*	.001
		top2^{deg}	.932
5b	*top2*^{deg} *top2-B44*	*top2*^{deg}	.000
		top2^{deg} *top2*^{Y782F}	.002
		top2^{deg} *top2-B44*^{Y782F}	.003
5b	*top2*^{deg}	*top2*^{deg} *top2-B44*	.000
		top2^{deg} *top2*^{Y782F}	.812
		top2^{deg} *top2-B44*^{Y782F}	.621
5b	*top2*^{deg} *top2*^{Y782F}	*top2*^{deg} *top2-B44*	.002
		top2^{deg}	.812
		top2^{deg} *top2-B44*^{Y782F}	.985
5b	*top2*^{deg} *top2-B44*^{Y782F}	*top2*^{deg} *top2-B44*	.003
		top2^{deg}	.621
		top2^{deg} *top2*^{Y782F}	.985
6c	*top2*^{deg} *top2-B44*	*top2*^{deg}	.011
		top2^{deg} *top2*^{G144I}	.924
6c	*top2*^{deg}	*top2*^{deg} *top2-B44*	.011
		top2^{deg} *top2*^{G144I}	.017
6c	*top2*^{deg} *top2*^{G144I}	*top2*^{deg} *top2-B44*	.924
		top2^{deg}	.017

Furniss et al. Supplemental Table 1

Figure	Strain 1	Strain 2	Significance
6d	*top2^{deg}*	*top2^{deg} top2^{G144I}*	.000
		top2^{deg} top2-G144I mad2Δ	.987
6d	*top2^{deg} top2^{G144I}*	*top2^{deg}*	.000
		top2^{deg} top2-G144I mad2Δ	.000
6d	*top2^{deg} top2-G144I mad2Δ*	*top2^{deg}*	.987
		top2^{deg} top2^{G144I}	.000
6g	*top2^{deg}*	*top2^{deg} top2^{G144I}*	.001
		top2^{deg} top2^{G144I} rad53-1	.001
6g	*top2^{deg} top2^{G144I}*	*top2^{deg}*	.001
		top2^{deg} top2^{G144I} rad53-1	.997
6g	*top2^{deg} top2^{G144I} rad53-1*	*top2^{deg}*	.001
		top2^{deg} top2^{G144I}	.997
6f	*top2^{deg} top2-B44*	*top2^{deg}*	.016
		top2^{deg} top2^{E66Q}	.479
6f	*top2^{deg}*	*top2^{deg} top2-B44*	.016
		top2^{deg} top2^{E66Q}	.083
6f	*top2^{deg} top2^{E66Q}*	*top2^{deg} top2-B44*	.479
		top2^{deg}	.086
6e	*top2^{deg}*	*top2^{deg} top2^{E66Q}*	.034
		top2^{deg} top2^{E66Q} mad2Δ	.943
6e	*top2^{deg} top2^{E66Q}*	*top2^{deg}*	.034
		top2^{deg} top2^{E66Q} mad2Δ	.015
6e	*top2^{deg} top2^{E66Q} mad2Δ*	*top2^{deg}*	.943
		top2^{deg} top2^{E66Q}	.015
6h	*top2^{deg} rad53-1*	*top2^{deg} top2^{E66Q}*	.016
		top2^{deg} top2^{E66Q} rad53-1	.000
6h	*top2^{deg} top2^{E66Q}*	*top2^{deg} rad53-1*	.016
		top2^{deg} top2^{E66Q} rad53-1	.051
6h	*top2^{deg} top2^{E66Q} rad53-1*	*top2^{deg} rad53-1*	.000
		top2^{deg} top2^{E66Q}	.051

Furniss et al. Supplemental Table 1

Figure	Strain 1	Strain 2	Significance
7b	*top2*^{deg} *top2-B44*	*top2*^{deg}	.000
		top2^{deg} *top2*$^{L475A/L480P}$	.468
7b	*top2*^{deg}	*top2*^{deg} *top2-B44*	.000
		top2^{deg} *top2*$^{L475A/L480P}$	.000
7b	*top2*^{deg} *top2*$^{L475A/L480P}$	*top2*^{deg} *top2-B44*	.468
		top2^{deg}	.000
7c	*top2*^{deg}	*top2*^{deg} *top2*$^{L475A/L480}$	.004
		top2^{deg} *top2*$^{L475A/L480}$ *mad2Δ*	.759
7c	*top2*^{deg} *top2*$^{L475A/L480}$	*top2*^{deg}	.004
		top2^{deg} *top2*$^{L475A/L480}$ *mad2Δ*	.001
7c	*top2*^{deg} *top2*$^{L475A/L480}$ *mad2Δ*	*top2*^{deg}	.759
		top2^{deg} *top2*$^{L475A/L480}$	.001
7d	*top2*^{deg} *rad53-1*	*top2*^{deg} *top2*$^{L475A/L480}$	.000
		top2^{deg} *top2*$^{L475A/L480}$ *rad53-1*	.000
7d	*top2*^{deg} *top2*$^{L475A/L480}$	*top2*^{deg} *rad53-1*	.000
		top2^{deg} *top2*$^{L475A/L480}$ *rad53-1*	.067
7d	*top2*^{deg} *top2*$^{L475A/L480}$ *rad53-1*	*top2*^{deg} *rad53-1*	.000
		top2^{deg} *top2*$^{L475A/L480}$	.067
8a	*top2*^{deg} *top2-B44*	*top2*^{deg}	.006
		top2^{deg} *top2*^{G738D}	.006
8a	*top2*^{deg}	*top2*^{deg} *top2-B44*	.006
		top2^{deg} *top2*^{G738D}	.999
8a	*top2*^{deg} *top2*^{G738D}	*top2*^{deg} *top2-B44*	.006
		top2^{deg}	.999
8a	*top2*^{deg} *top2-B44*	*top2*^{deg}	.000
		top2^{deg} *top2*^{P824S}	.000
8a	*top2*^{deg}	*top2*^{deg} *top2-B44*	.000
		top2^{deg} *top2*^{P824S}	.864
8a	*top2*^{deg} *top2*^{P824S}	*top2*^{deg} *top2-B44*	.000
		top2^{deg}	.864

Furniss et al. Supplemental Table 1
