# Supplementary material for: Direct Monitoring of the Strand Passage Reaction of DNA Topoisomerase II Triggers Checkpoint Activation
Source: PLoS Genet. 2013 Oct 3;9(10):e1003832. doi: 10.1371/journal.pgen.1003832 (PMC3789831; doi:10.1371/journal.pgen.1003832)
Supplement: Table S2 — Yeast strains used in this study. (PDF) [file pgen.1003832.s014.pdf]

Table S2

Yeast strains used in this study.

Strain	Number	Genotype
top2^{deg}	4075	*MATa bar1Δ top2::KAN^{R} leu2::Met3-Ub-DHFR-HA-TOP2 (LEU2) Gal-myc-UBR1 (HIS3) ura3::HIS3:Tub1-GFP (URA3) pRS414 (TRP1)*
top2^{deg} TOP2	4051	*MATa bar1Δ top2::KAN^{R} leu2::Met3-Ub-DHFR-HA-TOP2 (LEU2) Gal-myc-UBR1 (HIS3) ura3::HIS3:Tub1-GFP (URA3) pRS414-TOP2 (TRP1)*
top2^{deg} top2-B44	4053	*MATa bar1Δ top2::KAN^{R} leu2::Met3-Ub-DHFR-HA-TOP2 (LEU2) Gal-myc-UBR1 (HIS3) ura3::HIS3:Tub1-GFP (URA3) pRS414-top2-B44 (TRP1)*
top2^{deg} top2-B44 mad2Δ	4081	*MATa bar1Δ top2::KAN leu2::Met3-Ub-DHFR-HA-TOP2 (LEU2) Gal-myc-UBR1 (HIS3) ura3::HIS3:Tub1-GFP (URA3) mad2::KAN pRS414-top2-B44 (TRP1)*
top2^{deg} top2-B44^{K651A}	4269	*MATa bar1Δ top2::KAN^{R} leu2::Met3-Ub-DHFR-HA-TOP2 (LEU2) Gal-myc-UBR1 (HIS3) ura3::HIS3:Tub1-GFP (URA3) pRS414-top2-B44^{K651A} (TRP1)*
top2^{deg} top2^{Y782F}	4284	*MATa bar1Δ top2::KAN^{R} leu2::Met3-Ub-DHFR-HA-TOP2 (LEU2) Gal-myc-UBR1 (HIS3) ura3::HIS3:Tub1-GFP (URA3) pRS414-top2^{Y782F} (TRP1)*
top2^{deg} top2-B44^{Y782F}	4285	*MATa bar1Δ top2::KAN^{R} leu2::Met3-Ub-DHFR-HA-TOP2 (LEU2) Gal-myc-UBR1 (HIS3) ura3::HIS3:Tub1-GFP (URA3) pRS414-top2-B44^{Y782F} (TRP1)*
top2^{deg} top2^{G144I}	4276	*MATa bar1Δ top2::KAN^{R} leu2::Met3-Ub-DHFR-HA-TOP2 (LEU2) Gal-myc-UBR1 (HIS3) ura3::HIS3:Tub1-GFP (URA3) pRS414-top2^{G144I} (TRP1)*
top2^{deg} top2$^{G144I, Y782F}$	4355	*MATa bar1Δ top2::KAN^{R} leu2::Met3-Ub-DHFR-HA-TOP2 (LEU2) Gal-myc-UBR1 (HIS3) ura3::HIS3:Tub1-GFP (URA3) pRS414-top2$^{G144I , Y782F}$(TRP1)*
top2^{deg} top2^{G144I} mad2Δ	4359	*MATa bar1Δ top2::KAN leu2::Met3-Ub-DHFR-HA-TOP2 (LEU2) Gal-myc-UBR1 (HIS3) ura3::HIS3:Tub1-GFP (URA3) mad2::KAN pRS414-top2^{G144I} (TRP1)*
top2^{deg} top2^{G144I} rad53-1	4359	*MATa bar1Δ top2::KAN leu2::Met3-Ub-DHFR-HA-TOP2 (LEU2) Gal-myc-UBR1 (HIS3) ura3::HIS3:Tub1-GFP (URA3) rad53-1 pRS414-top2^{G144I} (TRP1)*
top2^{deg} top2^{E66Q}	4330	*MATa bar1Δ top2::KAN^{R} leu2::Met3-Ub-DHFR-HA-TOP2 (LEU2) Gal-myc-UBR1 (HIS3) ura3::HIS3:Tub1-GFP (URA3) pRS414-top2^{E66Q} (TRP1)*
top2^{deg} top2^{E66Q} mad2Δ	KFY147	*MATa bar1Δ top2::KAN leu2::Met3-Ub-DHFR-HA-TOP2 (LEU2) Gal-myc-UBR1 (HIS3) ura3::HIS3:Tub1-GFP (URA3) mad2::KAN pRS414-top2^{E66Q} (TRP1)*
top2^{deg} top2^{E66Q} rad53-1	4357	*MATa bar1Δ top2::KAN leu2::Met3-Ub-DHFR-HA-TOP2 (LEU2) Gal-myc-UBR1 (HIS3) ura3::HIS3:Tub1-GFP (URA3) rad53-1 pRS414-top2^{G144I} (TRP1)*
top2^{deg} rad53-1	4383	*MATa bar1Δ top2::KAN leu2::Met3-Ub-DHFR-HA-TOP2 (LEU2) Gal-myc-UBR1 (HIS3) ura3::HIS3:Tub1-GFP (URA3) rad53-1 pRS414 (TOP2)*
top2^{deg} top2$^{L475A/L480P}$	4331	*MATa bar1Δ top2::KAN^{R} leu2::Met3-Ub-DHFR-HA-TOP2 (LEU2) Gal-myc-UBR1 (HIS3) ura3::HIS3:Tub1-GFP (URA3) pRS414-top2$^{L475A/L480P}$ (TRP1)*
top2^{deg} top2$^{L475A/L480P}$ mad2Δ	KFY148	*MATa bar1Δ top2::KAN leu2::Met3-Ub-DHFR-HA-TOP2 (LEU2) Gal-myc-UBR1 (HIS3) ura3::HIS3:Tub1-GFP (URA3) mad2::KAN pRS414-top2$^{L475A/L480P}$ (TRP1)*
top2^{deg} top2$^{L475A/L480P}$ rad53-1	4385	*MATa bar1Δ top2::KAN leu2::Met3-Ub-DHFR-HA-TOP2 (LEU2) Gal-myc-UBR1 (HIS3) ura3::HIS3:Tub1-GFP (URA3) rad53-1 pRS414-top2$^{L475A/L480P}$ (TRP1)*
top2^{deg} top2^{G738D}	4277	*MATa bar1Δ top2::KAN^{R} leu2::Met3-Ub-DHFR-HA-TOP2 (LEU2) Gal-myc-UBR1 (HIS3) ura3::HIS3:Tub1-GFP (URA3) pRS414-top2^{G738D} (TRP1)*
top2^{deg} top2^{P824S}	4279	*MATa bar1Δ top2::KAN^{R} leu2::Met3-Ub-DHFR-HA-TOP2 (LEU2) Gal-myc-UBR1 (HIS3) ura3::HIS3:Tub1-GFP (URA3) pRS414-top2^{P824S} (TRP1)*

Furniss et al. Supplemental Table 2

Strain	Number	Genotype
rad53Δ top2^deg^	4536	*MATa bar1Δ top2::KAN^R^ leu2::Met3-Ub-DHFR-HA-TOP2 (LEU2) Gal-myc-UBR1 (HIS3) ura3::HIS3:Tub1-GFP pRS414-RNR1(TRP1)*
TOP2 rad53Δ top2^deg^	4537	*MATa bar1Δ top2::KAN^R^ leu2::Met3-Ub-DHFR-HA-TOP2 (LEU2) Gal-myc-UBR1 (HIS3) ura3::HIS3:Tub1-GFP rad53::KAN pRS414-RNR1(TRP1) YCp50-TOP2(URA3)*
top2-B44 rad53Δ top2^deg^	4538	*MATa bar1Δ top2::KAN^R^ leu2::Met3-Ub-DHFR-HA-TOP2 (LEU2) Gal-myc-UBR1 (HIS3) ura3::HIS3:Tub1-GFP rad53::KAN pRS414-RNR1(TRP1) YCp50-top2-B44(URA3)*
top2-Y782F rad53Δ top2^deg^	4539	*MATa bar1Δ top2::KAN^R^ leu2::Met3-Ub-DHFR-HA-TOP2 (LEU2) Gal-myc-UBR1 (HIS3) ura3::HIS3:Tub1-GFP rad53::KAN pRS414-RNR1(TRP1) YCp50-top2-Y782F(URA3)*
top2-G144I rad53Δ top2^deg^	4540	*MATa bar1Δ top2::KAN^R^ leu2::Met3-Ub-DHFR-HA-TOP2 (LEU2) Gal-myc-UBR1 (HIS3) ura3::HIS3:Tub1-GFP rad53::KAN pRS414-RNR1(TRP1) YCp50-top2-G144I(URA3)*
top2-E66Q rad53Δ top2^deg^	4541	*MATa bar1Δ top2::KAN^R^ leu2::Met3-Ub-DHFR-HA-TOP2 (LEU2) Gal-myc-UBR1 (HIS3) ura3::HIS3:Tub1-GFP rad53::KAN pRS414-RNR1(TRP1) YCp50-top2-E66Q(URA3)*
top2-L475A/L480P rad53Δ top2^deg^	4542	*MATa bar1Δ top2::KAN^R^ leu2::Met3-Ub-DHFR-HA-TOP2 (LEU2) Gal-myc-UBR1 (HIS3) ura3::HIS3:Tub1-GFP rad53::KAN pRS414-RNR1(TRP1) YCp50-top2-L475A/L480P(URA3)*
TOP2 RAD53-Flag top2^deg^	4551	*MATa bar1Δ top2::KAN^R^ leu2::Met3-Ub-DHFR-HA-TOP2 (LEU2) Gal-myc-UBR1 (HIS3) ura3::HIS3:Tub1-GFP RAD53-FLAG(TRP1) YCp50-TOP2(URA3)*
top2-B44 RAD53-Flag top2^deg^	4552	*MATa bar1Δ top2::KAN^R^ leu2::Met3-Ub-DHFR-HA-TOP2 (LEU2) Gal-myc-UBR1 (HIS3) ura3::HIS3:Tub1-GFP RAD53-FLAG(TRP1) YCp50-top2-B44(URA3)*
top2-Y782F RAD53-Flag top2^deg^	4553	*MATa bar1Δ top2::KAN^R^ leu2::Met3-Ub-DHFR-HA-TOP2 (LEU2) Gal-myc-UBR1 (HIS3) ura3::HIS3:Tub1-GFP RAD53-FLAG(TRP1) YCp50-top2-Y782F(URA3)*
top2-G144I RAD53-Flag top2^deg^	4554	*MATa bar1Δ top2::KAN^R^ leu2::Met3-Ub-DHFR-HA-TOP2 (LEU2) Gal-myc-UBR1 (HIS3) ura3::HIS3:Tub1-GFP RAD53-FLAG(TRP1) YCp50-top2-G144I(URA3)*
top2-E66Q RAD53-Flag top2^deg^	4555	*MATa bar1Δ top2::KAN^R^ leu2::Met3-Ub-DHFR-HA-TOP2 (LEU2) Gal-myc-UBR1 (HIS3) ura3::HIS3:Tub1-GFP RAD53-FLAG(TRP1) YCp50-top2-E66Q(URA3)*
top2-L475A/L480P RAD53-Flag top2^deg^	4556	*MATa bar1Δ top2::KAN^R^ leu2::Met3-Ub-DHFR-HA-TOP2 (LEU2) Gal-myc-UBR1 (HIS3) ura3::HIS3:Tub1-GFP RAD53-FLAG(TRP1) YCp50-top2-L475A/L480P(URA3)*
top2^deg^ top2-B44ΔCTR	4244	*MATa bar1Δ top2::KAN^R^ leu2::Met3-Ub-DHFR-HA-TOP2 (LEU2) Gal-myc-UBR1 (HIS3) ura3::HIS3:Tub1-GFP (URA3) pRS414-top2-B44ΔCTR (TRP1)*
top2^deg^ top2-B44 GAL-CTR	4251	*MATa bar1Δ top2::KAN leu2::Met3-Ub-DHFR-HA-TOP2 (LEU2) Gal-myc-UBR1 (HIS3) ura3::HIS3:Tub1-GFP pRS414-top2-B44 (TRP1) pGAL-CTR (URA3)*
MAD2-GFP top2-B44	4514	*MATa bar1Δ top2-B44 MAD2-3xGFP::KAN*
MAD2-GFP	4516	*MATa bar1Δ MAD2-3xGFP::KAN*
ndc10-1	3098	*MATa bar1Δ ndc10-1 ura3::HIS3:Tub1-GFP*
ndc10-1 top2-B44	4354	*MATa bar1Δ ndc10-1 top2-B44 ura3::HIS3:Tub1-GFP*

Furniss et al. Supplemental Table 2 (continued)
